# Microglial-expressed genetic risk variants, cognitive function and brain volume in patients with schizophrenia and healthy controls

**DOI:** 10.1038/s41398-021-01616-z

**Published:** 2021-09-23

**Authors:** Emma Corley, Laurena Holleran, Laura Fahey, Aiden Corvin, Derek W. Morris, Gary Donohoe

**Affiliations:** 1 School of Psychology, National University of Ireland, Galway, Ireland; 2 Centre for Neuroimaging and Cognitive Genomics, National University of Ireland Galway, Galway, Ireland; 3 Discipline of Biochemistry, National University of Ireland Galway, Galway, Ireland; 4 Neuropsychiatric Genetics Research Group, Department of Psychiatry, Institute of Molecular Medicine, Trinity College Dublin, Dublin, Ireland

**Keywords:** Molecular neuroscience, Psychiatric disorders

## Abstract

Changes in immune function are associated with variance in cognitive functioning in schizophrenia. Given that microglia are the primary innate immune cells in the brain, we examined whether schizophrenia risk-associated microglial genes (measured via polygenic score analysis) explained variation in cognition in patients with schizophrenia and controls (*n* = 1,238) and tested whether grey matter mediated this association. We further sought to replicate these associations in an independent sample of UK Biobank participants (*n* = 134,827). We then compared the strength of these microglial associations to that of neuronal and astroglial (i.e., other brain-expressed genes) polygenic scores, and used MAGMA to test for enrichment of these gene-sets with schizophrenia risk. Increased microglial schizophrenia polygenic risk was associated with significantly lower performance across several measures of cognitive functioning in both samples; associations which were then found to be mediated via total grey matter volume in the UK Biobank. Unlike neuronal genes which did show evidence of enrichment, the microglial gene-set was not significantly enriched for schizophrenia, suggesting that the relevance of microglia may be for neurodevelopmental processes related more generally to cognition. Further, the microglial polygenic score was associated with performance on a range of cognitive measures in a manner comparable to the neuronal schizophrenia polygenic score, with fewer cognitive associations observed for the astroglial score. In conclusion, our study supports the growing evidence of the importance of immune processes to understanding cognition and brain structure in both patients and in the healthy population.

## Introduction

Schizophrenia (SZ) is a complex neuropsychiatric disorder in which the level of disability is strongly predicted by impairments in cognitive function. Although the aetiology of SZ remains only partly understood, liability is genetically mediated with complex environmental interactions involved. Genome-wide association studies (GWAS) have demonstrated that the immune system may be involved in these interactions, largely due to the strong association of genetic variants in the major histocompatibility complex (MHC) [1, 2]. In humans, the MHC region encodes ~230 genes and is associated with immune function. In particular, variation of the complement component 4 (C4) gene has been associated with SZ risk [3]; we recently demonstrated that genetically predicted higher C4 expression was associated with both reduced memory function and reduced cortical activation [4]. We have further shown that these cognitive and cortical associations appear to generalise to genetic risk variants in the complement pathway more broadly [5, 6]. Complement protein expression has also been linked with an increased risk of transition from a high-risk state to psychosis [7].

In addition to evidence associating the complement pathway to SZ risk and cognition, microglia—the resident macrophages of the brain—have been associated with SZ pathophysiology and cognition. These cells have several functions including, but not limited to, phagocytosing apoptotic cells, synaptic pruning, modulating neurogenesis, and regulating synapse plasticity [8, 9]. Given these roles, altered microglia function has been hypothesised to influence a variety of aspects of brain physiology and cognitive function relevant to SZ [10]. This is supported by findings of elevated peripheral cytokines such as IL-6, IL-1β, TNF-α and C-reactive protein in patients [11, 12], which have in turn been linked to reductions in grey matter (GM) volume and with poorer cognitive performance [13]. In addition, post-mortem and in vivo neuroimaging studies have reported aberrant microglial activation in SZ, particularly in the hippocampal area, a region synonymous with memory function and dysfunction [14–17]. However, whether microglial perturbations are genetically mediated by structural brain measures has not yet been established.

Given the link between the immune system and cognition, we sought to investigate the effects of a microglial genetic score on cognitive performance and total GM in a sample (*n* = 1,238) of patients with SZ and healthy participants (discovery sample). In addition to considering any issues of multiple testing, our rationale for selecting this global metric of brain structure was based on this region being robustly associated with cognitive ability. Further, to offset any potential issues of power, we replicated these associations in a large independent sample of UK Biobank participants (*n* = 134,827). We hypothesised that participants carrying a higher SZ polygenic burden would demonstrate poorer performance on measures of cognitive ability and that lower total GM volume would mediate this relationship. We further hypothesised that microglial genes would be enriched for SZ risk and that microglial PGS would demonstrate an association with cognitive ability in a manner comparable to other brain-relevant genetic variants previously implicated in SZ, cognition and/or immunity (i.e., neurons and astroglia). Such investigations into genetic and structural imaging correlates of cognition may help elucidate a potential mechanism by which immune genes contribute to the cognitive deficits observed in SZ.

## Materials and methods

### Irish discovery sample

A total of 1,238 Irish SZ patients and controls (489 females, 740 males) aged 18–72 (mean = 41.15, s.d. = 12.84) were included in this study for whom neuropsychological data and genome-wide data were available. These consisted of *n* = 908 clinically stable patients with either (a) a diagnosis of SZ and schizoaffective disorder (SZA) (*n* = 676) and a broader category of psychosis cases diagnosed with either (1) bipolar disorder with psychotic features, (2) major depressive disorder with psychotic features, (3) delusional disorder and (4) psychosis not otherwise specified (*n* = 232). To ascertain a diagnosis, patients were administered the Structural Clinical Interview for the DSM-IV [18]. Healthy participants (*n* = 330) were recruited from the general population through local media advertisements and online. They were aged between 18 and 65 years and had no history of major mental health issues, intellectual disability or acquired brain injury. Further information on this sample has been detailed elsewhere [4, 6, 19]^.^ All assessments carried out were in accordance with the relevant ethics committees’ approval from each participating site and informed written consent was obtained prior to the study. Participants undergoing structural MRI were also screened for MRI safety criteria.

### Cognitive assessment

Cognitive functioning was examined across three domains including general cognitive ability, working memory and episodic memory. General cognitive functioning was measured using selected subtests from the Wechsler Adult Intelligence Scale, third edition (WAIS-III) [20] and the Wechsler Test of Adult Reading (WTAR) [21]. Working memory was assessed using the LetterNumber Sequencing (LNS) task from the Wechsler Memory Scale, third edition (WMS-III) [22] and the Spatial Working Memory task from the Cambridge Automated Neuropsychological Test Battery (CANTAB) [23]. Episodic memory was examined using the logical memory subtests and the faces subtests (immediate and delayed conditions) from the WMS-III, as well as the paired associations learning task (PAL; stages completed and total errors) from the CANTAB. To reduce multiple testing burden, an unrotated principal component analysis was performed for the available episodic memory tests and explained 72% of the variance in memory scores.

### Structural MRI

A subset of participants (47 patients, 66 healthy participants) underwent structural MRI. MR images were obtained on a Philips Intera Achieva 3 T MR system, with whole-brain imaging consisting of T1-weighted images (180 slices-duration 6 min) using a turbo field echo gradient pulse sequence, with a slice thickness of 0.9 mm and a 230 × 230 field of view. Detailed procedures for the image volumetric analysis have been described elsewhere [19]. Briefly, cortical reconstruction, parcellation and segmentation of the T1 images were processed using FreeSurfer (v6.0) [24]. Processed images underwent motion correction, intensity normalisation, transformation to Talairach space and skull stripping. Whole-hemisphere measures were visually inspected and statistically evaluated for outliers following standardised ENIGMA protocols (http://enigma.ini.usc.edu/protocols/imaging-protocols).

### Genetic data

Genotype data were obtained from DNA extracted from saliva or whole blood samples. Full GWAS data were available on all samples. Participants for this study had been genotyped with either an Affymetrix 6.0 chip as part of the Wellcome Trust Case Control Consortium 2 [25] or on the Illumina HumanCoreExome chip. Imputation of these data sets was performed using 1000 Genomes Phase I data and IMPUTE2 [26] to yield approximately 10 million single nucleotide polymorphisms (SNPs) genome-wide per sample. Only SNPs that passed QC filtering were imputed using the 1000 Genomes reference panel.

#### Brain expressed gene-sets

To explore the impact of SZ risk alleles linked to microglia, we restricted our SZ-PGS and gene-set analysis to loci expressed within microglial cells. This microglial list (*n* = 294) was based on recent transcriptome work by Saunders et al. [27], which used Drop-seq to profile single-cell RNA expression in 690,000 cells and 565 cell types from the adult mouse brain, accessible through the online software *DropViz* (http://dropviz.org/). For this, we selected genes that demonstrated greater than two-fold increased expression within microglial cells. Mouse gene IDs were converted to human gene IDs using BioMart.

**Fig. 1:**
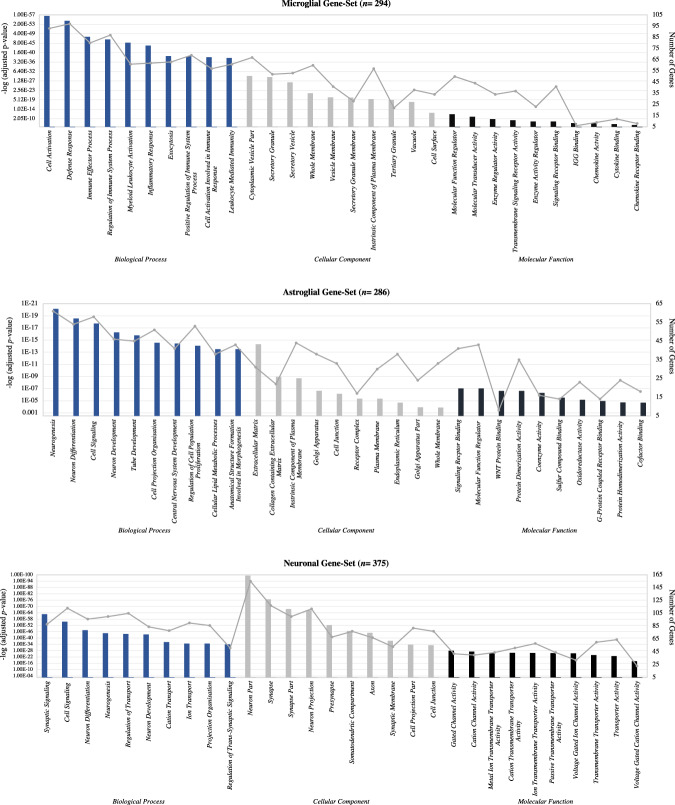
Gene ontology enrichment analysis. GO enrichment analysis for the top ten biological processes (BPs), cellular components (CCs) and molecular functions (MFs) of the microglial, neuronal and astroglial gene-sets. Analyses indicated that these gene-sets were good representations of each cell types specific functioning. The line plot displays the numbers of differentially expressed genes in each specific function. The bar plot displays the adjusted *p* values (FDR (false discovery rate) < 0.05).

To compare these microglial genes with other brain-relevant genes, we generated two additional gene-sets from genes expressed within neuronal (*n* = 375) and astroglial (*n* = 286) cells; again, based on genes showing greater than two-fold increased expression. The rationale for choosing these other gene-sets is as follows: (1) cognitive functioning is largely dependent on the expression of neuronal genes and (2) astroglial cells represent the other cell type that is most consistently associated with neuroinflammatory processes (see Supplementary Tables 1–3 for details of these gene-sets). As a complementary analysis, GO enrichment of biological processes, cellular components and molecular functions was performed using the software FUMA [28] to test if processes associated with specific cell type functions were uniquely enriched within each gene-set. As shown in Fig. 1, this analysis confirmed that the microglial, neuronal and astroglial gene-sets were good representatives of each cell type-specific functioning. A schematic overview of the steps taken for generating these gene sets is shown in Fig. S1, along with the subsequent analytical procedures.

#### Polygenic score (PGS) calculation

SZ-PGSs were calculated based on the results from the SZ GWAS meta-analysis conducted on 40,675 cases and 64,643 controls of European ancestry [2]. For each of the gene-sets, we identified SNPs within our genes of interest and captured SNPs within and outside the gene’s coding region by specifying a symmetrical annotation window of ±20 kb. Genotype data for SNPs in these gene-set regions were extracted for our Irish samples from their full GWAS data. PLINK 1.9 software was used to perform quality control (QC) on the data [29] whereby SNPs were excluded if minor allele frequency (MAF) < 0.1%, SNP missingness ≤2% and Hardy–Weinberg equilibrium < P10-6. An effect-size weighted SZ-PGS for the microglia, neurons and astroglia variants were then computed for each individual based on a threshold of *p* < 0.05 using PRSice2 software [30]. A *p*-value threshold of 0.05 was chosen since this has been shown to maximally capture polygenic risk across a large number of independent samples [1]. Variants within the MHC (chr6:25–35 Mb) were removed from the genotype data due to high linkage disequilibrium in this region.

#### Gene enrichment analysis

For our gene-set analyses, we used MAGMA v1.06 [31] to test if the microglial, neuronal and astroglial gene-sets were enriched for genes associated with SZ risk. Briefly, enrichment analyses consisted of the following steps: (1) an annotation step to map SNPs onto genes; (2) a gene analysis step to identify gene *p*-values using the most recent SZ GWAS summary statistics [2]; and (3) a gene-level analysis to test whether these gene cell types showed enrichment for SZ.

### Statistical analysis

To estimate microglial SZ-PGS effects on cognitive performance, multiple regression analyses were carried out using IBM SPSS Statistics Version 26.0 (IBM Corp, Armonk, NY, 2017), with cognitive test scores as the dependent variable. The significance threshold was set at *p* < 0.016 to correct for the 3 domains of cognitive function (*α* = 0.05/3 = 0.016). To examine if total GM volume mediated the association between PGS and cognitive performance we used model 4 from the PROCESS v3.5 macro [32]. A bootstrapping method was applied to assess the indirect effects based on 10,000 bootstrapped samples using 95% bias-corrected confidence intervals. Microglial SZ-PGS associations were then compared to neuronal and astroglial SZ-PGSs by deriving a *Z* score based on the statistical significance of the associations found and their effect size (standardised beta value). We did so to determine whether SZ-associated SNPs within microglial genes showed a stronger effect on cognitive performance than other selected brain-expressed genes. Throughout, all analyses were corrected for age and sex and intracranial volume was controlled for in our imaging analysis.

### UK biobank replication sample

To examine whether the results for SZ-PGS predicting cognition in patients and controls were comparable with results in a population-based sample, we tested these SZ-PGS associations in an independent dataset of UK Biobank participants (*n* = 134,827). The analytic methods followed those of the main case–control analysis using the discovery Irish sample (cognitive measures: fluid intelligence score, numerical memory score, symbol-digit substitution score and general cognitive ability (constructed using a PCA of the three available cognitive tests)). UK Biobank analyses were conducted under project number 23739 and approved by the National Health Service Research Ethics Service (reference 11/NW/0382). For details of the imaging, genotyping, QC and imputation, see Supplementary Information.

## Results

### Participant characteristics of the Irish discovery and UK biobank sample

Participant characteristics of the discovery sample, including clinical, demographic and cognitive information are presented in Table S4 (see Table S5 for UK Biobank participant details). Pearson’s product-moment correlation coefficients were first carried out to test an association between the SZ-PGSs and covariates of no interest. Results indicated no association between the SZ-PGSs and age, sex, or years in education in either sample. Further, in our discovery sample of patients, no associations between the SZ-PGSs, the severity of symptoms or medication dosage (as measured in terms of chlorpromazine equivalents) were found.

### Association between microglial SZ-polygenic scores and cognition

For the regression analyses, which included the full discovery sample, microglial SZ-PGS significantly predicted variation in performance IQ (*F* change = 7.727, *R*^2^ change = 0.009, Std beta = −0.096, *p* = 0.006), full-scale IQ (*F* change = 6.618, *R*^*2*^ change = 0.008, Std beta = −0.089, *p* = 0.010) and episodic memory (*F* change = 13.703; *R*^2^ change = 0.018, Std beta = −0.135, *p* < 0.001) as well as nominal associations with verbal IQ and the LNS task (Table 1). The observed direction of effect was that greater microglial SZ-PGS was associated with a decrease in cognitive performance.

**Table 1:** Regression analysis of polygenic scores and cognitive performance across gene-sets in the discovery and UK Biobank sample.

		Microglia				Neurons				Astroglia		
	*F* change	*R*^2^ change	*β*	*p*	*F* change	*R*^2^ change	*β*	*p*	*F* change	*R*^2^ change	*β*	*p*
*Discovery sample*												
Full-scale IQ	6.62	0.008	−0.089	0.010	13.17	0.015	−0.124	<0.001	1.05	0.001	−0.038	0.306
Performance IQ	7.73	0.009	−0.096	0.006	11.61	0.013	−0.116	0.001	2.46	0.003	−0.058	0.117
Verbal IQ	4.78	0.005	−0.070	0.029	10.38	0.010	−0.101	0.001	0.44	0.001	−0.024	0.507
WTAR	1.84	0.002	−0.047	0.176	7.56	0.009	−0.095	0.006	0.01	0.001	0.000	0.909
LNS	5.47	0.006	−0.075	0.020	0.52	0.001	−0.023	0.472	4.37	0.006	−0.078	0.037
Spatial WM	2.37	0.003	−0.058	0.124	3.81	0.005	−0.073	0.051	0.63	0.001	−0.032	0.427
Episodic memory	13.70	0.018	−0.135	<0.001	8.17	0.011	−0.104	0.004	4.95	0.008	−0.087	0.027
*UKB replication*												
Fluid intelligence	21.08	0.00018	−0.013	<0.001	30.73	0.000263	−0.016	<0.001	19.44	0.00016	−0.013	<0.001
Numerical memory	0.405	0.00001	−0.003	0.525	0.098	0.000002	0.002	0.755	0.24	0.000006	−0.002	0.627
Symbol-digit	19.05	0.00036	−0.019	<0.001	21.26	0.000400	−0.012	<0.001	11.22	0.00021	−0.015	0.001
General cognition	5.45	0.00016	−0.013	0.020	4.77	0.000143	−0.013	0.029	2.98	0.00009	−0.009	0.084

*WTAR* Wechsler test of adult reading, *LNS* letter-number sequencing, *Spatial WM* spatial working memory.

The most markedly significant domain was found for episodic memory, and when the sample was subdivided into patients and controls, this association remained significant in cases (*F* change = 4.842, *R*^2^ change = 0.009, Std beta = −0.096, *p* = 0.028) and trended toward significance in controls (*F* change = 3.68, *R*^2^ change = 0.024, Std beta = −0.154, *p* = 0.057). In a post hoc analysis of narrow psychosis patients (SZ and SZA patients only), the association between the microglial PGS and episodic memory was in the same direction (*F* change = 1.217, *R*^2^ change = 0.003, Std beta = −0.055) but was nonsignificant (*p* = 0.271). All other cognitive measures though did not show any significant association with PGS at either nominal or corrected significance level when grouped accordingly. Details of these group comparisons are reported in Supplementary Table 6.

### Association between microglia, cognition and GM volume

**Fig. 2:**
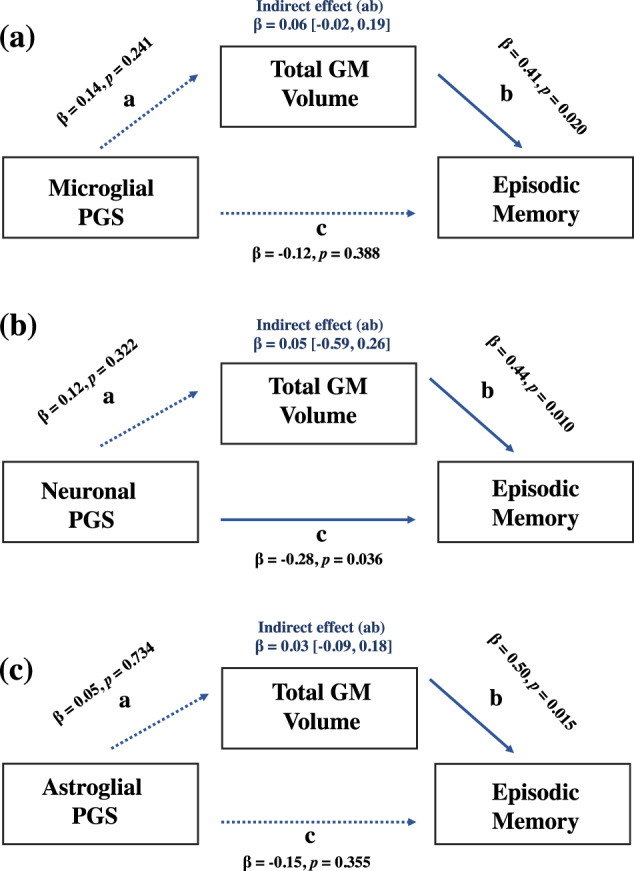
Mediation results of polygenic score associations in the discovery sample. Values are standardised estimates; dashed paths are nonsignificant. Indirect estimates are reported (a*b). **a** The effect of the microglial polygenic score on episodic memory as mediated via grey matter volume. **b** The effect of the neuronal polygenic score on episodic memory as mediated via grey matter volume. **c** The effect of the astroglial polygenic score on episodic memory as mediated via grey matter volume.

Given the significant association between microglial SZ-PGS and episodic memory, we sought to investigate whether a possible interaction existed between microglia and brain structure, as indexed by total GM volume. For this, no significant interaction effect between the microglial SZ-PGS and GM volume for episodic memory was observed in the sample. The standardised indirect effect was 0.0572 and the 95% bias-corrected CIs were −0.0237 to 0.193 (Fig. 2). However, the total number of participants with complete genetic and MRI overlap for this memory test was small (*n* = 47) and the non-significance of this association could reflect the limited power of the sample.

### A cognitive comparison of microglia, neuronal and astroglial SZ-polygenic scores

When comparing the effect size estimates of the different SZ-PGSs, the difference in magnitude between the microglial, neuronal and astroglial SZ-PGSs with cognitive ability was not statistically significant (Table 2). However, the association between neuronal SZ-PGS and cognitive ability was comparable to the microglial SZ-PGS, which significantly predicted variation across multiple measures of cognition (*R*^2^ change range = 0.009–0.015, Std beta range = −0.095 to −0.124). By comparison, only nominal significant weak associations were observed between astroglial SZ-PGS and two cognitive measures—episodic memory (Std beta = −0.087, *p* = 0.027) and the LNS task (Std beta = −0.078, *p* = 0.037) (Table 1). For the mediation analysis, GM volume did not mediate the association between these SZ-PGSs and episodic memory (Fig. 2).

**Table 2:** Cognitive comparison of the microglia, neuronal and astroglial polygenic scores.

	Microglia vs. neurons		Neurons vs. astroglia		Microglia vs. astroglia	
	*Z*-score	*p*-Value	*Z-*score	*p-*Value	*Z*-score	*p*-Value
*Discovery sample*						
Full-Scale IQ	0.69	0.490	1.63	0.103	0.96	0.337
Performance IQ	0.40	0.689	1.11	0.267	0.72	0.472
Verbal IQ	0.67	0.503	1.54	0.124	0.91	0.363
WTAR	0.96	0.337	−1.83	0.067	−0.59	0.374
LNS	1.12	0.268	1.09	0.276	0.06	0.952
Spatial WM	0.281	0.790	0.75	0.453	0.47	0.638
Episodic Memory	0.56	0.576	0.30	0.764	0.84	0.401
*UKB replication*						
Fluid Intelligence	0.72	0.472	0.72	0.472	0	1
Numerical Memory	−0.71	0.478	−0.57	0.569	0.14	0.889
Symbol-Digit	1.03	0.303	0.44	0.660	0.59	0.555
General Cognition	0	1	0.49	0.624	0.49	0.624

*WTAR* Wechsler test of adult reading, *LNS* letter-number sequencing, *Spatial WM* spatial working memory.

### UK biobank replication sample

For the regression analyses which included UK Biobank participants, microglial SZ-PGS significantly predicted variation in fluid intelligence (*F* change = 21.084, *R*^2^ change = 0.0002, Std beta = −0.013, *p* < 0.001) and the symbol digit substitution test (*F* change = 19.047, *R*^2^ change = 0.0004, Std beta = −0.019, *p*  < 0.001) but not numerical memory (*F* change = 0.405, *R*^2^ change = 0.00001, Std beta = −0.003, *p* = 0.525) (Table 1). After performing an unrotated principal component of these cognitive tests, this combined general cognitive ability factor was significantly associated with microglial SZ-PGS (*F* change = 5.451, *R*^2^ change = 0.0002, Std beta = −0.013, *p* = 0.020). The direction of these effects was the same as those observed for our discovery sample (i.e., increased SZ-PGS was associated with lower cognitive ability). The neuronal and astrocyte SZ-PGSs also significantly predicted variation in cognitive performance (Table 1), although again the association between astroglial SZ-PGS and cognitive function was weaker than for the other two gene sets.

**Fig. 3:**
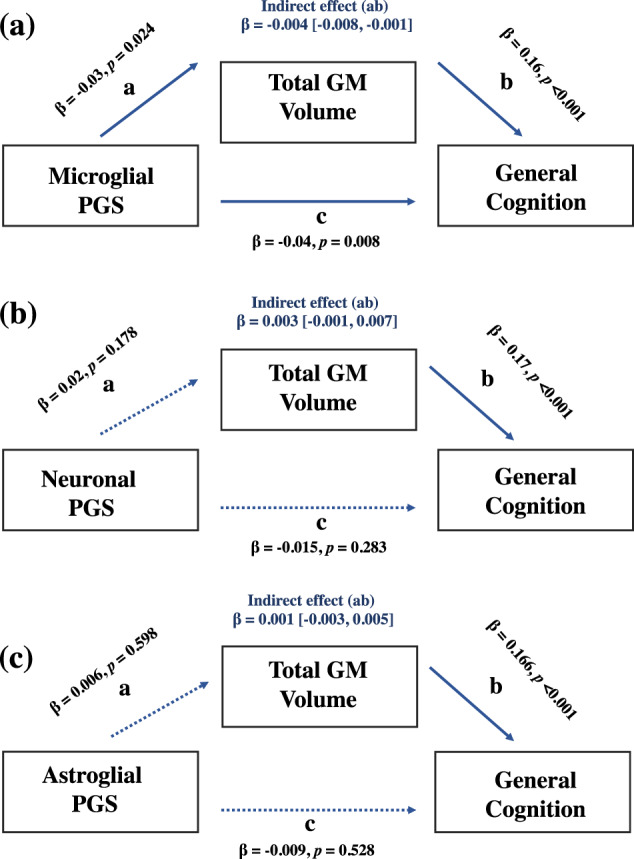
Mediation results of polygenic score associations in the UK Biobank sample. Values are standardised estimates; dashed paths are nonsignificant. Indirect estimates are reported (a*b). **a** The effect of the microglial polygenic score on general cognitive ability as mediated via grey matter volume. **b** The effect of the neuronal polygenic score on general cognitive ability as mediated via grey matter volume. **c** The effect of the astroglial polygenic score on general cognitive as mediated via grey matter volume.

To examine whether these associations were mediated via GM volume, we selected general cognitive ability, rather than memory performance given the non-significant SZ-PGSs associations with this test. Here, we found that total GM volume partially mediated the relationship between the microglial SZ-PGS and general cognitive ability. As shown in Fig. 3, the standardised regression coefficient for GM volume and cognitive ability and for microglial SZ-PGS and cognitive ability were both significant. The standardised indirect effect was −0.0042 (95% CI based on 10,000 samples: −0.0081 to −0.0005). In contrast, GM volume did not significantly mediate the SZ-PGS cognitive association for either neuronal (std beta = 0.0026, 95% CIs [−0.0012 to 0.0065], *p* = 0.285) or astroglial (std beta = 0.0010, 95% CIs [−0.0027 to 0.0047], *p* = 0.528) expressed genes (Fig. 3). Consistent with the results from the discovery sample, effect size estimates for the SZ-PGS associations with measures of cognitive functioning were not statistically different across gene sets (Table 2).

### Enrichment analysis

We tested for enrichment of microglial, neuronal and astroglial gene-sets with risk of SZ, controlling for a number of SNPs in each set. For this analysis, genotype information was available for 203 microglial genes, 350 neuronal genes and 262 astroglial genes after excluding missing/incomplete SNP data. Here, we found that the microglial gene-set was not significantly enriched for genes associated with SZ risk (std beta = −0.0087, SE = 0.0773, *p* = 0.860). Similarly, no enrichment was observed for the astroglial gene-set (std beta= 0.004, SE = 0.0662, *p* = 0.268). By comparison, the neuronal gene-set showed enrichment for SZ risk (std beta = −0.0285, SE = 0.058, *p* = 0.0002).

## Discussion

In this study, we sought to characterise the effects of microglial-expressed genetic variants on cognitive functioning and brain structure in a sample of patients with SZ and healthy participants. To our knowledge, this is the first genetic study to associate variation in a microglial-expressed gene-set to cognitive performance. Consistent with previous studies showing a link between complement genes and cognition carried out by us [4–6, 33] and others [34, 35], we found that a microglial SZ-PGS predicted variation on multiple cognitive measures, with the strongest association observed for memory function. In our replication sample, we further found evidence that total GM volume mediated the association between microglial SZ-PGS and cognitive ability. Remarkably, while the effect size estimates between the different PGSs and cognition did not differ significantly, the microglial SZ-PGS was predictive of cognitive functioning to a comparable extent as found for the neuronal SZ-PGS. This suggests that, in addition to neuronal genes, microglial-expressed common genetic variation may also have relevance to cognitive functioning, both in patients and in healthy participants, and to understanding brain structural development related to cognition.

### Polygenic score analysis of the microglial gene-set

In the regression analysis of the discovery sample, increased microglial SZ-PGS was associated with lower scores on several cognitive measures (performance IQ, full-scale IQ and episodic memory). While these associations were observed for a range of neurocognitive functions, the cognitive domain most strongly associated with the microglial SZ-PGS was episodic memory. These findings are highly consistent with previous studies documenting an association between immune genetic risk variants and memory performance in patients. As noted above, we have previously demonstrated an association between CSMD1 (a gene implicated in complement function) and memory [33] in patients with SZ, and further shown that genetically predicted C4 RNA expression correlates with episodic memory performance [4]. Among the cognitive impairments associated with SZ, deficits in memory function are consistently amongst the largest observed [36]. It is interesting to speculate about why memory function is associated with immune-related genetic variation—whether this simply reflects the size of deficit associated with SZ, or an aspect of cognition sensitive to either immune function or factors related to immune function (e.g. stress). While association data by its nature prevents us from drawing any causal influences, it is noteworthy that the association observed was not specific to patients, but rather observed across the entire sample, suggesting that this association may not be illness-specific.

In addition to SZ, microglial abnormalities have also been found to influence memory performance in several different cohorts, including in patients with Alzheimer’s Disease (AD) [37]. Interestingly, in AD, microglial overactivation has been found to co-occur with structural changes in the brain and with memory deficits [38]. Although few studies have directly examined the relationship between microglial pathology and brain structure in SZ, these findings are noteworthy here, as they suggest that brain structure and cognitive processes may be linked to microglial abnormalities. Interestingly, despite the absence of a direct relationship between microglia and brain structure, our study found that GM volume mediated the association between microglial PGS and general cognitive ability. At a genetic level, this suggests that microglia may associate with structural brain changes indirectly via cognitive functioning. However, this mediation effect was only observed in the UK Biobank sample, possibly reflecting a false-positive finding or a lack of power in the smaller discovery sample. As such, confirmation of these results will require testing in large longitudinal datasets consisting of genetic, cognitive and imaging data.

### Comparison of the microglial, neuronal and astroglial polygenic scores

When examining other gene cell types expressed in the brain, we found that the neuronal SZ-PGS was, as expected, also significantly associated with measures of cognitive performance. Indeed, neuronal susceptibility variants have consistently been implicated in SZ and in the cognitive symptoms of the disorder (e.g., Lips et al. [39]; Hertzberg et al. [40]). In our study, we found that the neuronal SZ-PGS showed effects with cognitive performance that were not specific to any one aspect of cognition. This may suggest that neuronally expressed genes have a more general impact on cognitive functions in SZ, unlike microglial genes which showed a stronger influence on memory processes. By contrast, the astroglial SZ-PGS predicted variation in memory performance at a nominal level only, explaining considerably less variance on this aspect of cognition compared to the microglial and neuronal SZ-PGSs. Finally, unlike neuronal genes, our microglial gene-set did not show evidence of enrichment for SZ risk. This, together with the significant associations of the microglial PGS analysis, may indicate that the relevance of microglial-expressed genetic variation is for neurodevelopmental processes related more generally to cognition, rather than to illness risk per se.

### Strengths and limitations

Strengths of our study include a novel investigation of the relationship between microglial genetic variants and cognition, the relatively large size of our case/control sample and replication of these findings in a larger cohort. At the same time, the impact of the microglial SZ-PGS on cognition was small (0.8–1.8% of the variance explained). Although modest, this is consistent with prior research demonstrating that associations between common variants and SZ risk and cognition are of small effect. In addition, the total number of individuals with complete genetic, cognitive and MRI data in our discovery sample was small, limiting our ability to infer an effect of GM volume on microglia and cognition, particularly in the patient group. Further, the microglial gene-set was derived from mouse brain data- the caveat being the non-identity of genetic variation and brain morphology to humans (e.g., immune-relevant genes such as C4A, C4B and SIGEC-11 are not present in mice but they are in humans [3, 41]). We also acknowledge that although genes were selected based on their expression levels within certain cell types, many genes may have alternative roles in immune and non-immune functions. However, our enrichment analysis for GO biological processes and molecular functions supports that these gene-sets are to a large extent good representatives of the cell types specific functioning (Fig. 1). A final limitation is that our SZ-PGSs were derived using relatively common SNPs (MAF > 0.01) and did not include rare SNPs identified via exome-based studies, although SNPs with lower frequencies are unlikely to greatly affect the group-wide findings reported here.

## Conclusion

In conclusion, our findings provide evidence that increased microglial polygenic risk is associated with decreased performance in both patients with SZ and healthy controls. We further highlight that variation in GM volume may mediate the observed association between the microglial PGS and cognition, supporting evidence of immune processes being associated with variation in brain structure. We also highlight, both the non-illness specificity of the findings and the absence of microglial enrichment for SZ risk and interpret this to reflect the relevance of microglial-expressed genetic variation is for neurodevelopmental processes related more generally to cognition. This is also consistent with the finding that the strength of association between the microglial and neuronal PGSs did not differ significantly but rather demonstrated comparable associations across the various cognitive tests.

Notwithstanding, future studies with larger SZ samples will be required to clarify whether illness-specific effects exist between microglia, cognition and brain structure. Further, giving the growing evidence that not just genetic but also environmental factors (e.g., childhood adversity) are associated with immune function and cognition in SZ [42–44], modelling the interaction between immune-related genetic variants and environmental factors will be an important avenue for future research. Finally, modelling the association between microglial function and cognition in animal studies will be necessary for eliciting the neural basis of the association reported here.

## Supplementary information

Supplementary Information
