# Supplementary material for: Microglial-expressed genetic risk variants, cognitive function and brain volume in patients with schizophrenia and healthy controls
Source: Transl Psychiatry. 2021 Sep 23;11:490. doi: 10.1038/s41398-021-01616-z (PMC8460789; doi:10.1038/s41398-021-01616-z)
Supplement: Supplementary file 1 — Supplementary Information [file 41398_2021_1616_MOESM1_ESM.docx]

**Supplemental Information**

**Microglial-Expressed Genetic Risk Variants, Cognitive function and Brain Volume in Patients with Schizophrenia and Healthy Controls**

**Authors:** Emma Corley^a,b^, Laurena Holleran^a,b^, Laura Fahey^b,c,^, Aiden Corvin^d^, Derek W. Morris^b,c^, Gary Donohoe^a,b*^

**Affiliations:**

^a^ School of Psychology, National University of Ireland Galway, Ireland.

^b^ Centre for Neuroimaging and Cognitive Genomics, National University of Ireland Galway, Ireland.

^c^ Discipline of Biochemistry, National University of Ireland Galway, Ireland.

^d^ Neuropsychiatric Genetics Research Group, Department of Psychiatry, Institute of Molecular Medicine, Trinity College Dublin, Dublin, Ireland.

***Corresponding author:**

*Prof. Gary Donohoe*

*School of Psychology & Centre for Neuroimaging and Cognitive Genomics*

*National University of Ireland Galway, Ireland*

*Tel: +353 91 49 5122; Email: gary.donohoe@nuigalway.ie*

**UK Biobank Sample**

**Descriptive Statistics of UK Biobank Sample**

A total of 134,827 UK Biobank participants (71,843 females, 62,984 males) aged 40-73 years (mean= 56.38, s.d.= 7.69) had cognitive and genome-wide data available.

**UK Biobank Cognitive Measures**

The three cognitive tests used in the present study were fluid intelligence (UK Biobank Field ID: 20016), symbol-digit substitution (UK Biobank Field ID: 20159) and numerical memory (UK Biobank Field ID: 20240). The fluid intelligence test consisted of a series of 13 items assessing verbal and arithmetical deduction (Cronbach α reliability= 0.62)^1^. The symbol-digit test, which is similar in format to the Symbol Digit Modalities Test^2^ involved matching symbols to single-digit integers. The score was based on the number of correct symbol-digit matches made in 60 seconds. For the numerical memory test participants were shown a two-digit number which they had to recall after a short pause. Numbers increased by one until the participant made an error or until they reached the maximum number of 12 digits. As a measure of general cognitive ability, scores of these three cognitive tests were entered into an unrotated principal component analysis. This intelligence factor explained 51% of variance in intelligence scores.

**UK Biobank Structural MRI Data**

MRI data were collected in a single Siemens Skyra 3T scanner with a standard 32-channel head coil located at UK Biobank’s recruitment centre. T1-weighted MPRAGE data was acquired in the sagittal plane using a three-dimensional magnetization-prepared rapid gradient-echo sequence at a resolution of 1 x 1 x 1 mm, with a 208 x 256 x 256 field of view. Global and regional brain IPDs were extracted using FMRIB’s Automated Segmentation Tool (FAST)^3^. A global brain imaging-derived phenotype of total grey matter volume (UK Biobank field ID: 25006) was used. Participants with severe and visual normalisation problems were removed by the UK Biobank. Further details of the brain imaging protocols have been published elsewhere^4^.

**Genotype Data**

Genotyping was performed using the Affymetrix UK BiLEVE Axiom array (807,411 probes) on 50,000 individuals, and the Affymetrix UK Biobank Axiom ® array (820,967 probes) on the rest of the sample. The two arrays have over 95% common content. Before the release of the genetic data, quality control (QC) measures were applied and details of these steps can be found in Bycroft et al.,^5^. Building on these QC metrics, we excluded SNPs on the basis of SNP missingness > 0.02, minor allele frequency (MAF) <0.01, Hardy–Weinberg equilibrium (HWE) ⩽ 1 × 10^-6^, imputation quality score <0.9 and differing allele frequency between the two arrays. PLINK 1.9 software was used to perform quality control (QC) on the data^6^. Individuals were also removed based on non-European ancestry, relatedness, discordant sex information, high heterozygosity/missingness, chromosomal aneuploidies and retracted consent. Following the QC steps described above, 64,478 SNPs were included for analysis.

**Supplementary Table 1 |** List of microglial Expressed Genes, including their Chromosome and Base Pair Locations (± 20 KB)

| Gene Name | Chromosome | Start | Stop |
| --- | --- | --- | --- |
| ABCA9 | 17 | 66950773 | 67078442 |
| ADAM17 | 2 | 9609392 | 9715917 |
| ADAP2 | 17 | 29228698 | 29306340 |
| ADGRE1 | 19 | 6867560 | 6960464 |
| ADORA3 | 1 | 112005970 | 112126602 |
| ADRB2 | 5 | 148186156 | 148274628 |
| AIF1 | 6 | 31562969 | 31604798 |
| AIM2 | 1 | 159008790 | 159066685 |
| ALOX5AP | 13 | 31267615 | 31358565 |
| ANG | 14 | 21132336 | 21182345 |
| APBB1IP | 10 | 26707253 | 26876732 |
| ARHGAP17 | 16 | 24910710 | 25046695 |
| ARHGAP30 | 1 | 160996731 | 161059760 |
| ARHGDIB | 12 | 15074949 | 15134562 |
| ATF3 | 1 | 212718676 | 212814119 |
| B4GALT1 | 9 | 33090636 | 33187356 |
| BANK1 | 4 | 102691764 | 103015969 |
| BASP1 | 5 | 17196932 | 17296954 |
| BIN2 | 12 | 51654822 | 51738446 |
| BLNK | 10 | 97931455 | 98051333 |
| BLVRB | 19 | 40933691 | 40991725 |
| BMP2K | 4 | 79677532 | 79857519 |
| BST2 | 19 | 17482238 | 17536458 |
| C15orf39 | 15 | 75471219 | 75524510 |
| C1QA | 1 | 22943118 | 22986175 |
| C1QB | 1 | 22959682 | 23008130 |
| C1QC | 1 | 22949969 | 22994603 |
| C3AR1 | 12 | 8190919 | 8238955 |
| C4A | 6 | 31929834 | 31990457 |
| C4B | 6 | 31962572 | 32023195 |
| C5AR1 | 19 | 47793104 | 47845327 |
| CCDC88B | 11 | 64087690 | 64145006 |
| CCL15 | 17 | 34303476 | 34349084 |
| CCL18 | 17 | 34371643 | 34418841 |
| CCL23 | 17 | 34320096 | 34365005 |
| CCL24 | 7 | 75420766 | 75472674 |
| CCL3 | 17 | 34395602 | 34437506 |
| CCL3L1 | 17 | 34603842 | 34645730 |
| CCL4 | 17 | 34411220 | 34453014 |
| CCL4L2 | 17 | 34518468 | 34560275 |
| CCL8 | 17 | 32626066 | 32668421 |
| CCR5 | 3 | 46391633 | 46437697 |
| CD14 | 5 | 139991313 | 140033286 |
| CD163 | 12 | 7603412 | 7676414 |
| CD180 | 5 | 66457205 | 66512617 |
| CD300A | 17 | 72442509 | 72500937 |
| CD300LD | 17 | 72556111 | 72608370 |
| CD302 | 2 | 160605139 | 160675115 |
| CD33 | 19 | 51708335 | 51763274 |
| CD36 | 7 | 80211504 | 80328593 |
| CD37 | 19 | 49818632 | 49863863 |
| CD52 | 1 | 26624411 | 26667014 |
| CD53 | 1 | 111393821 | 111462558 |
| CD68 | 17 | 7462805 | 7505429 |
| CD74 | 5 | 149761200 | 149812543 |
| CD84 | 1 | 160490884 | 160569306 |
| CD86 | 3 | 121754209 | 121859990 |
| CEBPA | 19 | 33770840 | 33813430 |
| CFH | 1 | 196601008 | 196736634 |
| CFP | X | 47463612 | 47509704 |
| CLEC10A | 17 | 6957856 | 7003626 |
| CLEC4A | 12 | 8256228 | 8311203 |
| CLEC4C | 12 | 7860235 | 7922069 |
| CLEC4M | 19 | 7808035 | 7854491 |
| CLEC5A | 7 | 141607157 | 141666783 |
| CLEC6A | 12 | 8588591 | 8650926 |
| CMTM6 | 3 | 32502804 | 32564403 |
| COL27A1 | 9 | 116898231 | 117092975 |
| CPA3 | 3 | 148563043 | 148634874 |
| CRYBB1 | 22 | 26975362 | 27033991 |
| CSF1R | 5 | 149412854 | 149512935 |
| CSF3R | 1 | 36911644 | 36968915 |
| CTC1 | 17 | 8108139 | 8171413 |
| CTSC | 11 | 88006760 | 88090941 |
| CTSH | 15 | 79194092 | 79257436 |
| CTSS | 1 | 150682672 | 150758433 |
| CX3CR1 | 3 | 39284985 | 39343226 |
| CYBA | 16 | 88689697 | 88737492 |
| CYBB | X | 37619266 | 37692718 |
| CYSLTR1 | X | 77506965 | 77603193 |
| CYTH4 | 22 | 37658495 | 37731389 |
| DAB2 | 5 | 39351776 | 39445335 |
| DHRS3 | 1 | 12607939 | 12697820 |
| DNASE2 | 19 | 12966025 | 13012409 |
| DOCK2 | 5 | 169044251 | 169530386 |
| DOCK8 | 9 | 194865 | 485259 |
| DSE | 6 | 116581231 | 116782422 |
| EDEM1 | 3 | 5209359 | 5281650 |
| ELK3 | 12 | 96568207 | 96681606 |
| EMP3 | 19 | 48808629 | 48853810 |
| ENTPD1 | 10 | 97451536 | 97657023 |
| F11R | 1 | 160945001 | 161011133 |
| F13A1 | 6 | 6124311 | 6340924 |
| FAM111A | 11 | 58890219 | 58942512 |
| FCER1G | 1 | 161165087 | 161209038 |
| FCGR1A | 1 | 149734232 | 149784074 |
| FCGR1B | 1 | 120906128 | 120955944 |
| FCGR2A | 1 | 161455205 | 161509360 |
| FCGR2B | 1 | 161612905 | 161668444 |
| FCGR2C | 1 | 161531129 | 161591010 |
| FCRL2 | 1 | 157695523 | 157767119 |
| FCRL3 | 1 | 157626271 | 157690775 |
| FERMT3 | 11 | 63954152 | 64011363 |
| FES | 15 | 91407665 | 91459006 |
| FGD2 | 6 | 36953423 | 37016845 |
| FLI1 | 11 | 128536430 | 128703162 |
| FMNL1 | 17 | 43279192 | 43344685 |
| FMNL3 | 12 | 50011724 | 50121197 |
| FOLR2 | 11 | 71907819 | 71952994 |
| GAL3ST4 | 7 | 99736865 | 99786373 |
| GBP3 | 1 | 89452360 | 89508556 |
| GCNT1 | 9 | 79014752 | 79142332 |
| GMFG | 19 | 39798999 | 39846726 |
| GMIP | 19 | 19720285 | 19774457 |
| GNA15 | 19 | 3116191 | 3183766 |
| GNGT2 | 17 | 47263596 | 47307936 |
| GOLM1 | 9 | 88609018 | 88735116 |
| GPR183 | 13 | 99926789 | 99979749 |
| GPR34 | X | 41528221 | 41576530 |
| GPSM3 | 6 | 32138543 | 32183300 |
| GPX3 | 5 | 150379999 | 150428554 |
| GRAP | 17 | 18903969 | 18970336 |
| GRAPL | 17 | 19010782 | 19082148 |
| GSDMD | 8 | 144615383 | 144665232 |
| GUSB | 7 | 65405671 | 65467301 |
| HACD4 | 9 | 20983620 | 21051635 |
| HAVCR2 | 5 | 156492843 | 156556248 |
| HCK | 20 | 30619991 | 30709659 |
| HCLS1 | 3 | 121330246 | 121399791 |
| HHEX | 10 | 94429681 | 94475408 |
| HK2 | 2 | 75039782 | 75140481 |
| HLA-DQB1 | 6 | 32607241 | 32654466 |
| HLA-DQB2 | 6 | 32703837 | 32751330 |
| HPGD | 4 | 175391328 | 175464049 |
| HPGDS | 4 | 95199707 | 95284027 |
| IFI27L2 | 14 | 94574118 | 94615957 |
| IFI30 | 19 | 18264590 | 18308934 |
| IGF1 | 12 | 102769645 | 102895563 |
| IGFBP4 | 17 | 38579676 | 38633982 |
| IL10RA | 11 | 117837106 | 117892198 |
| IL10RB | 21 | 34618665 | 34689539 |
| IL16 | 15 | 81454941 | 81625104 |
| IL21R | 16 | 27393483 | 27483363 |
| IL6R | 1 | 154357669 | 154461926 |
| IRF5 | 7 | 128557976 | 128610089 |
| IRF8 | 16 | 85912774 | 85976212 |
| ITGAM | 16 | 31251288 | 31364213 |
| ITGB2 | 21 | 46285864 | 46368753 |
| ITGB5 | 3 | 124460795 | 124626500 |
| ITPR2 | 12 | 26468285 | 27006131 |
| KCNK6 | 19 | 38790484 | 38839654 |
| LAIR1 | 19 | 54845235 | 54902241 |
| LAPTM5 | 1 | 31185315 | 31250683 |
| LAT2 | 7 | 73604087 | 73664164 |
| LCP1 | 13 | 46680058 | 46776459 |
| LCP2 | 5 | 169655088 | 169744822 |
| LGALS13 | 19 | 40073169 | 40118114 |
| LGALS14 | 19 | 40174946 | 40220088 |
| LGALS16 | 19 | 40126558 | 40171287 |
| LGALS3BP | 17 | 76947335 | 76996061 |
| LHFPL2 | 5 | 77761038 | 77964648 |
| LIMD2 | 17 | 61753249 | 61798527 |
| LIPA | 10 | 90953326 | 91031660 |
| LPAR6 | 13 | 48965181 | 49038840 |
| LPCAT2 | 16 | 55522913 | 55640582 |
| LRRC3 | 21 | 45855393 | 45898739 |
| LSP1 | 11 | 1854200 | 1933493 |
| LST1 | 6 | 31533956 | 31576686 |
| LTC4S | 5 | 179199263 | 179243616 |
| LY86 | 6 | 6568934 | 6675216 |
| LYL1 | 19 | 13188002 | 13233974 |
| LYN | 8 | 56772386 | 56945006 |
| LYVE1 | 11 | 10558712 | 10610365 |
| LYZ | 12 | 69722134 | 69768013 |
| MAF | 16 | 79607745 | 79654622 |
| MAFB | 20 | 39294488 | 39337880 |
| MAN1C1 | 1 | 25923959 | 26131258 |
| MAN2B1 | 19 | 12737322 | 12797591 |
| MLXIPL | 7 | 72987524 | 73058903 |
| MMP9 | 20 | 44617547 | 44665200 |
| MNDA | 1 | 158781168 | 158839270 |
| MPEG1 | 11 | 58955983 | 59000494 |
| MRC1 | 10 | 18078332 | 18220091 |
| MS4A6A | 11 | 59919080 | 59972139 |
| MS4A6E | 11 | 60082355 | 60128441 |
| MS4A7 | 11 | 60125949 | 60183427 |
| MYLIP | 6 | 16109277 | 16168479 |
| MYO1F | 19 | 8565674 | 8662331 |
| NAGPA | 16 | 5054845 | 5103942 |
| NCF1 | 7 | 74168309 | 74223720 |
| NCF2 | 1 | 183504697 | 183580056 |
| NCKAP1L | 12 | 54871495 | 54956899 |
| NRROS | 3 | 196346656 | 196408875 |
| OLFML3 | 1 | 114502030 | 114544875 |
| P2RX7 | 12 | 121550622 | 121644439 |
| P2RY12 | 3 | 151034631 | 151122600 |
| P2RY13 | 3 | 151024096 | 151067337 |
| P2RY6 | 11 | 72955550 | 73029670 |
| PAG1 | 8 | 81860045 | 82044303 |
| PALD1 | 10 | 72218564 | 72348206 |
| PDE3B | 11 | 14645191 | 14913605 |
| PF4 | 4 | 74826542 | 74867841 |
| PIK3CG | 7 | 106485723 | 106567592 |
| PLA2G15 | 16 | 68259240 | 68314965 |
| PLEK | 2 | 68572322 | 68644585 |
| PLEKHO1 | 1 | 150102170 | 150151825 |
| PLOD3 | 7 | 100829258 | 100881011 |
| PLXNB2 | 22 | 50693408 | 50766062 |
| PNP | 14 | 20917538 | 20966165 |
| PPCDC | 15 | 75295927 | 75363067 |
| PPFIA4 | 1 | 202975649 | 203067864 |
| PPP1R18 | 6 | 30624166 | 30675672 |
| PRCP | 11 | 82515409 | 82632733 |
| PRKCD | 3 | 53175223 | 53246733 |
| PROS1 | 3 | 93571881 | 93712934 |
| PSMB8 | 6 | 32788494 | 32832712 |
| PTAFR | 1 | 28453677 | 28540447 |
| PTGS1 | 9 | 125112809 | 125177982 |
| PTPN18 | 2 | 131093580 | 131152982 |
| PTPN6 | 12 | 7035740 | 7090479 |
| PTPRC | 1 | 198588098 | 198746605 |
| PYCARD | 16 | 31192807 | 31234097 |
| RAB3IL1 | 11 | 61644706 | 61733747 |
| RAC2 | 22 | 37601301 | 37660339 |
| RASAL3 | 19 | 15542435 | 15595382 |
| RCSD1 | 1 | 167579474 | 167697933 |
| RGS10 | 10 | 121239339 | 121322222 |
| RGS19 | 20 | 62684534 | 62731356 |
| RGS9 | 17 | 63113456 | 63243821 |
| RNASE4 | 14 | 21132259 | 21188761 |
| RNASEL | 1 | 182522769 | 182578420 |
| RNASET2 | 6 | 167322992 | 167390077 |
| RPS6KA1 | 1 | 26836249 | 26921520 |
| RUNX1 | 21 | 36140098 | 36441595 |
| SALL1 | 16 | 51149886 | 51205183 |
| SALL3 | 18 | 76720275 | 76779770 |
| SAMSN1 | 21 | 15837549 | 15975723 |
| SELPLG | 12 | 108995671 | 109047735 |
| SEMA4D | 9 | 91955706 | 92132906 |
| SFT2D2 | 1 | 168175255 | 168232088 |
| SH2B3 | 12 | 111823720 | 111909427 |
| SIGLEC6 | 19 | 52000951 | 52055110 |
| SIPA1 | 11 | 65385578 | 65438391 |
| SLA | 8 | 134028973 | 134135604 |
| SLC11A1 | 2 | 219226752 | 219281617 |
| SLC15A3 | 11 | 60684555 | 60739257 |
| SLC16A6 | 17 | 66243167 | 66307405 |
| SLC29A3 | 10 | 73059010 | 73143147 |
| SLC2A5 | 1 | 9077005 | 9151763 |
| SLC7A8 | 14 | 23574504 | 23672869 |
| SLC9A9 | 3 | 142964063 | 143587373 |
| SLCO2B1 | 11 | 74842032 | 74937445 |
| SLFN12 | 17 | 33718079 | 33780302 |
| SLFN12L | 17 | 33781942 | 33834758 |
| SLFN13 | 17 | 33742115 | 33795856 |
| SMAGP | 12 | 51619133 | 51684202 |
| SNX18 | 5 | 53793589 | 53862416 |
| SPI1 | 11 | 47356409 | 47420127 |
| STAB1 | 3 | 52509354 | 52578512 |
| STARD8 | X | 67847511 | 67965684 |
| STK17B | 2 | 196978307 | 197056336 |
| SULT1A1 | 16 | 28596908 | 28654907 |
| SUSD3 | 9 | 95800989 | 95867420 |
| SYNGR2 | 17 | 76144632 | 76189009 |
| TAC1 | 7 | 97341271 | 97389784 |
| TBXAS1 | 7 | 139458047 | 139740125 |
| TCIRG1 | 11 | 67786462 | 67838366 |
| TCN2 | 22 | 30983070 | 31043047 |
| TGFBI | 5 | 135344584 | 135419507 |
| TGFBR1 | 9 | 101847412 | 101936474 |
| TGFBR2 | 3 | 30627994 | 30755634 |
| TIFAB | 5 | 134764558 | 134808089 |
| TLR7 | X | 12865202 | 12928480 |
| TM6SF1 | 15 | 83756301 | 83826111 |
| TMEM106A | 17 | 41343865 | 41392061 |
| TMEM119 | 12 | 108963622 | 109011894 |
| TMEM173 | 5 | 138835113 | 138882343 |
| TMEM176A | 7 | 150477854 | 150522208 |
| TMEM37 | 2 | 120167501 | 120216096 |
| TMEM86A | 11 | 18700351 | 18746332 |
| TNFAIP8 | 5 | 118584418 | 118750294 |
| TNFAIP8L2 | 1 | 151109095 | 151152731 |
| TNFRSF1A | 12 | 6417923 | 6471283 |
| TREM2 | 6 | 41106244 | 41150924 |
| TRIM5 | 11 | 5656740 | 5726339 |
| TSPAN14 | 10 | 82194038 | 82302394 |
| TXNIP | 1 | 145418438 | 145462635 |
| TYROBP | 19 | 36375303 | 36419211 |
| UNC93B1 | 11 | 67738575 | 67791593 |
| VASP | 19 | 45990688 | 46050247 |
| VAV1 | 19 | 6752679 | 6877377 |
| WAS | X | 48522186 | 48569818 |
| ZFHX3 | 16 | 72796784 | 73112534 |
| ZFP36 | 19 | 39877487 | 39920052 |

**Supplementary Table 2 |** List of Neuronal Expressed Genes, including their Chromosome and Base Pair Locations (± 20 KB)

| Gene Name | Chromosome | Start | Stop |
| --- | --- | --- | --- |
| AAK1 | 2 | 69668532 | 69921481 |
| ABHD8 | 19 | 17382940 | 17441045 |
| ACHE | 7 | 100467615 | 100514594 |
| ADAM23 | 2 | 207288263 | 207505851 |
| ADARB1 | 21 | 46473768 | 46666478 |
| ADARB2 | 10 | 1208073 | 1799670 |
| ADCY1 | 7 | 45593739 | 45782715 |
| ADCYAP1 | 18 | 884944 | 932173 |
| ADD2 | 2 | 70814750 | 71015357 |
| AMIGO2 | 12 | 47449490 | 47493734 |
| ANKRD34B | 5 | 79832574 | 79886307 |
| ANO1 | 11 | 69904408 | 70055634 |
| ARHGAP20 | 11 | 110427766 | 110603912 |
| ARHGAP44 | 17 | 12672856 | 12914960 |
| ARHGEF17 | 11 | 72999334 | 73100136 |
| ARHGEF4 | 2 | 131574489 | 131824836 |
| ARL4C | 2 | 235381685 | 235425697 |
| ARX | X | 25001811 | 25054065 |
| ASIC4 | 2 | 220358892 | 220423494 |
| ATCAY | 19 | 3859862 | 3948077 |
| ATP1A3 | 19 | 42450734 | 42521649 |
| ATP2B2 | 3 | 10345707 | 10769716 |
| ATP6V1G2 | 6 | 31479694 | 31522076 |
| B3GALT2 | 1 | 193128175 | 193175784 |
| B4GALT6 | 18 | 29182210 | 29285799 |
| BAIAP3 | 16 | 1363602 | 1419439 |
| BCL11B | 14 | 99615624 | 99757861 |
| BCL2L15 | 1 | 114400790 | 114450169 |
| BEND6 | 6 | 56799773 | 56912140 |
| BEX1 | X | 102297579 | 102339168 |
| BEX2 | X | 102544274 | 102585974 |
| BTBD11 | 12 | 107692190 | 108073419 |
| C11orf87 | 11 | 109272846 | 109319840 |
| C1QTNF4 | 11 | 47591216 | 47636211 |
| CABP1 | 12 | 121058355 | 121125127 |
| CACNA1A | 19 | 13297256 | 13754804 |
| CACNA1B | 9 | 140752241 | 141039076 |
| CACNA1E | 1 | 181362238 | 181797219 |
| CACNA1G | 17 | 48618429 | 48724835 |
| CACNA2D1 | 7 | 81555760 | 82093114 |
| CACNA2D2 | 3 | 50380233 | 50561675 |
| CACNB3 | 12 | 49187577 | 49242724 |
| CADM3 | 1 | 159121399 | 159193103 |
| CADPS2 | 7 | 121938481 | 122546813 |
| CALB2 | 16 | 71372616 | 71444341 |
| CALN1 | 7 | 71224476 | 71932136 |
| CALY | 10 | 135118927 | 135170475 |
| CAMK1D | 10 | 12371481 | 12897545 |
| CAMK2A | 5 | 149579054 | 149689854 |
| CAMK2B | 7 | 44236749 | 44394176 |
| CAMK2N2 | 3 | 183957001 | 183999251 |
| CARTPT | 5 | 70994990 | 71036875 |
| CBLN2 | 18 | 70183915 | 70325756 |
| CD44 | 11 | 35140417 | 35273949 |
| CDK5R1 | 17 | 30793637 | 30838274 |
| CDS1 | 4 | 85484132 | 85592491 |
| CHAT | 10 | 50797141 | 50921925 |
| CHD5 | 1 | 6141853 | 6260183 |
| CHGA | 14 | 93369425 | 93421638 |
| CHGB | 20 | 5872076 | 5926007 |
| CHL1 | 3 | 218279 | 471090 |
| CHRM2 | 7 | 136533416 | 136725002 |
| CHRNA3 | 15 | 78865394 | 78933637 |
| CHRNB4 | 15 | 78896461 | 79040096 |
| CIB2 | 15 | 78376948 | 78443886 |
| CKMT1A | 15 | 43965084 | 44011420 |
| CKMT1B | 15 | 43865252 | 43917099 |
| CLEC2L | 7 | 139188602 | 139249730 |
| CLIP4 | 2 | 29300571 | 29432509 |
| CLSTN3 | 12 | 7262294 | 7331541 |
| CNR1 | 6 | 88829583 | 88896078 |
| CNTNAP1 | 17 | 40814631 | 40871832 |
| CNTNAP2 | 7 | 145793453 | 148138090 |
| CORO2A | 9 | 100863257 | 100974922 |
| CPLX1 | 4 | 758745 | 839986 |
| CPLX3 | 15 | 75098888 | 75144141 |
| CRH | 8 | 67068620 | 67110960 |
| CRMP1 | 4 | 5729811 | 5914785 |
| CX3CL1 | 16 | 57386370 | 57438960 |
| CXXC4 | 4 | 105369469 | 105436058 |
| CYGB | 17 | 74503438 | 74567257 |
| CYTIP | 2 | 158251131 | 158365473 |
| DACH1 | 13 | 71992098 | 72461330 |
| DCAF12L2 | X | 125278337 | 125320080 |
| DGKK | X | 50088408 | 50233737 |
| DGKQ | 4 | 932675 | 1000683 |
| DIRAS1 | 19 | 2694565 | 2741416 |
| DISP2 | 15 | 40630436 | 40683257 |
| DLG4 | 17 | 7073209 | 7143021 |
| DLK1 | 14 | 101172042 | 101221539 |
| DLX1 | 2 | 172929468 | 172974405 |
| DLX6 | 7 | 96614860 | 96660351 |
| DMTN | 8 | 21886506 | 21960038 |
| DMXL2 | 15 | 51719908 | 51935030 |
| DNAJC27 | 2 | 25146505 | 25214963 |
| DNM1 | 9 | 130945658 | 131037527 |
| DPP6 | 7 | 153564182 | 154705995 |
| DSCAM | 21 | 41362926 | 42239065 |
| DYNC1I1 | 7 | 95381866 | 95759634 |
| DYNC2H1 | 11 | 102960160 | 103370591 |
| DZANK1 | 20 | 18344011 | 18467925 |
| ECEL1 | 2 | 233324537 | 233372538 |
| EEF1A2 | 20 | 62099366 | 62150505 |
| EGFR | 7 | 55066714 | 55344313 |
| ELAVL2 | 9 | 23670102 | 23846335 |
| ELFN1 | 7 | 1707755 | 1807590 |
| ELMOD1 | 11 | 107441817 | 107557505 |
| ENO2 | 12 | 7002909 | 7052861 |
| ERBB4 | 2 | 212220446 | 213423565 |
| ERC2 | 3 | 55522336 | 56522391 |
| EVL | 14 | 100417786 | 100630573 |
| FAM155A | 13 | 107800883 | 108539083 |
| FAT2 | 5 | 150863654 | 150968505 |
| FIBCD1 | 9 | 133757825 | 133834673 |
| FOXP2 | 7 | 113706382 | 114353827 |
| FXYD6 | 11 | 117687693 | 117768201 |
| FZD1 | 7 | 90873783 | 90918123 |
| GABRA1 | 5 | 161254197 | 161346975 |
| GABRB3 | 15 | 26768693 | 27204686 |
| GABRG2 | 5 | 161474546 | 161602542 |
| GAD1 | 2 | 171649723 | 171737661 |
| GAD2 | 10 | 26485236 | 26613487 |
| GADD45A | 1 | 68130744 | 68174021 |
| GATA3 | 10 | 8075567 | 8137161 |
| GLRA1 | 5 | 151182074 | 151324403 |
| GNAL | 18 | 11668955 | 11905684 |
| GNAZ | 22 | 23392540 | 23487224 |
| GNG2 | 14 | 52272913 | 52466060 |
| GNG8 | 19 | 47117333 | 47157942 |
| GPR151 | 5 | 145872666 | 145915753 |
| GPR162 | 12 | 6910711 | 6959136 |
| GPR26 | 10 | 125405871 | 125474123 |
| GPRASP2 | X | 101947104 | 101993607 |
| GRIK1 | 21 | 30889254 | 31332351 |
| GRIN1 | 9 | 140012842 | 140083207 |
| GRIN2A | 16 | 9832376 | 10296611 |
| GRIN2B | 12 | 13673165 | 14153053 |
| GRIN2C | 17 | 72818162 | 72877627 |
| GRM1 | 6 | 146328782 | 146778734 |
| HAP1 | 17 | 39853994 | 39910896 |
| HAPLN4 | 19 | 19346450 | 19393605 |
| HECW1 | 7 | 43132198 | 43625600 |
| HOPX | 4 | 57494155 | 57568065 |
| HPCA | 1 | 33331595 | 33384042 |
| HS3ST4 | 16 | 25683347 | 26169009 |
| HS6ST2 | X | 131740044 | 132115423 |
| HSPA12A | 1 | 118410705 | 118522086 |
| HTR2C | X | 113798551 | 114164624 |
| HTR3A | 11 | 113825603 | 113881035 |
| IGHM | 14 | 106300349 | 106342323 |
| IPCEF1 | 6 | 154455631 | 154697926 |
| IQSEC3 | 12 | 96765 | 248460 |
| IRX1 | 5 | 3576168 | 3621517 |
| IRX2 | 5 | 2725959 | 2772969 |
| ISL1 | 5 | 50658921 | 50710564 |
| KCNAB2 | 1 | 6031526 | 6181253 |
| KCNC1 | 11 | 17736359 | 17824602 |
| KCNC3 | 19 | 50795194 | 50856772 |
| KCNG4 | 16 | 84235823 | 84293356 |
| KCNIP1 | 5 | 169760491 | 170183636 |
| KCNJ12 | 17 | 21259509 | 21343179 |
| KCNK2 | 1 | 215159118 | 215430436 |
| KCNK3 | 2 | 26895619 | 26976288 |
| KCNMA1 | 10 | 78609359 | 79418353 |
| KCNQ2 | 20 | 62017542 | 62123993 |
| KCTD8 | 4 | 44155926 | 44470824 |
| KIFC2 | 8 | 145671426 | 145719585 |
| KIT | 4 | 55504085 | 55626881 |
| KLC2 | 11 | 66004765 | 66055331 |
| KLHDC8B | 3 | 49189044 | 49233917 |
| KLHL1 | 13 | 70254726 | 70702591 |
| L1CAM | X | 153106969 | 153194677 |
| LGI2 | 4 | 24980469 | 25052501 |
| LHX1 | 17 | 35274084 | 35321917 |
| LHX6 | 9 | 124944856 | 125011905 |
| LHX8 | 1 | 75574119 | 75647218 |
| LINGO1 | 15 | 77885369 | 78133242 |
| LMTK3 | 19 | 48968528 | 49036446 |
| LONRF2 | 2 | 100869753 | 100959195 |
| LRFN5 | 14 | 42056773 | 42393752 |
| LRRC3B | 3 | 26644297 | 26772267 |
| LRRC55 | 11 | 56929221 | 56979191 |
| LRRTM3 | 10 | 68665764 | 68879588 |
| LY6H | 8 | 144219331 | 144262128 |
| LYNX1 | 8 | 143825752 | 143879640 |
| LYPD1 | 2 | 133382426 | 133449152 |
| MADD | 11 | 47270712 | 47371582 |
| MAPK10 | 4 | 86916276 | 87535284 |
| MAPK8 | 10 | 49494698 | 49667403 |
| MAPK8IP2 | 22 | 51019114 | 51072409 |
| MATK | 19 | 3757971 | 3822127 |
| MCTP1 | 5 | 94019446 | 94640279 |
| MEGF11 | 15 | 66167417 | 66578222 |
| MEIS2 | 15 | 37161406 | 37413504 |
| MTUS2 | 13 | 29578748 | 30097892 |
| MYT1L | 2 | 1772885 | 2355032 |
| NALCN | 13 | 101686130 | 102088843 |
| NAP1L2 | X | 72412135 | 72454684 |
| NAT8L | 4 | 2041239 | 2090816 |
| NCS1 | 9 | 132914857 | 133019583 |
| NDNF | 4 | 121936768 | 122014176 |
| NEFH | 22 | 29856219 | 29907379 |
| NETO2 | 16 | 47091614 | 47197908 |
| NEUROD1 | 2 | 182517815 | 182565603 |
| NGFR | 17 | 47552655 | 47612379 |
| NME7 | 1 | 169081769 | 169357205 |
| NMNAT2 | 1 | 183197372 | 183407737 |
| NOS1AP | 1 | 162019564 | 162373321 |
| NPPA | 1 | 11885766 | 11928402 |
| NPTX1 | 17 | 78420948 | 78471643 |
| NPTXR | 22 | 39194457 | 39259987 |
| NPY | 7 | 24303782 | 24351484 |
| NR4A2 | 2 | 157160944 | 157218860 |
| NRGN | 11 | 124589742 | 124637106 |
| NRIP3 | 11 | 8982123 | 9045596 |
| NRSN2 | 20 | 307426 | 360304 |
| NTRK1 | 1 | 156765432 | 156871642 |
| NXPH1 | 7 | 8453585 | 8812593 |
| NXPH3 | 17 | 47633220 | 47681189 |
| OGFOD1 | 16 | 56465402 | 56533012 |
| PDE11A | 2 | 178467980 | 178993066 |
| PDE1A | 2 | 182984763 | 183407919 |
| PDE1B | 12 | 54923134 | 54993023 |
| PDYN | 20 | 1939403 | 1994732 |
| PGM2L1 | 11 | 74021363 | 74129518 |
| PHYHIP | 8 | 22057222 | 22109854 |
| PKIA | 8 | 79408374 | 79537502 |
| PLCXD2 | 3 | 111373523 | 111585294 |
| PLD5 | 1 | 242226288 | 242707998 |
| PLEKHA6 | 1 | 204167979 | 204366793 |
| PLK5 | 19 | 1504073 | 1555455 |
| PLXNA4 | 7 | 131788091 | 132353447 |
| PMCH | 12 | 102570237 | 102611623 |
| PNCK | X | 152915185 | 152974465 |
| PNOC | 8 | 28154503 | 28220872 |
| PODXL2 | 3 | 127328024 | 127411652 |
| POU2F2 | 19 | 42570263 | 42720737 |
| POU4F1 | 13 | 79152497 | 79197673 |
| POU6F2 | 7 | 38997598 | 39552694 |
| PPP1R1B | 17 | 37762993 | 37812879 |
| PRKAR1B | 7 | 568834 | 787287 |
| PROX1 | 1 | 214136524 | 214234595 |
| PRRT2 | 16 | 29803177 | 29847201 |
| PRUNE2 | 9 | 79206292 | 79541003 |
| PSD | 10 | 104142376 | 104201296 |
| PTPN5 | 11 | 18729475 | 18834268 |
| PTPRT | 20 | 40681392 | 41838610 |
| PVALB | 22 | 37176728 | 37235523 |
| RAB3C | 5 | 57858048 | 58175213 |
| RALYL | 8 | 85075022 | 85854079 |
| RAPH1 | 2 | 204239068 | 204420133 |
| RASGEF1A | 10 | 43669983 | 43782367 |
| RASGRF1 | 15 | 79232289 | 79403115 |
| RBFOX1 | 16 | 6049095 | 7783340 |
| RBFOX3 | 17 | 77065427 | 77532230 |
| RBMS3 | 3 | 29302473 | 30071886 |
| REEP2 | 5 | 137754706 | 137802658 |
| RELN | 7 | 103092231 | 103649963 |
| RESP18 | 2 | 220172131 | 220217899 |
| RGS17 | 6 | 153305594 | 153472384 |
| RGS8 | 1 | 182595239 | 182673711 |
| RIMS1 | 6 | 72576406 | 73132845 |
| RIMS3 | 1 | 41066351 | 41151329 |
| RIT2 | 18 | 40303192 | 40715657 |
| RNF157 | 17 | 74118534 | 74256454 |
| ROBO2 | 3 | 75935846 | 77719115 |
| ROBO3 | 11 | 124715282 | 124771366 |
| RPH3A | 12 | 112988184 | 113356686 |
| RPRM | 2 | 154313852 | 154355322 |
| RSPO3 | 6 | 127419749 | 127538910 |
| RUSC1 | 1 | 155270687 | 155320905 |
| RYR2 | 1 | 237185505 | 238017288 |
| SACS | 13 | 23882965 | 24027841 |
| SAMD14 | 17 | 48167404 | 48227246 |
| SCG2 | 2 | 224441658 | 224487221 |
| SCN1A | 2 | 166825670 | 167004523 |
| SCN1B | 19 | 35501588 | 35551352 |
| SCN2B | 11 | 118012666 | 118067388 |
| SCN3A | 2 | 165924032 | 166080577 |
| SCN8A | 12 | 51964050 | 52226648 |
| SCN9A | 2 | 167031695 | 167252503 |
| SCRT1 | 8 | 145367965 | 145411184 |
| SCUBE1 | 22 | 43573289 | 43759394 |
| SEMA3E | 7 | 82973222 | 83298326 |
| SEMA6B | 19 | 4522600 | 4579820 |
| SERPINI1 | 3 | 167433031 | 167563356 |
| SH3BGR | 21 | 40797781 | 40907433 |
| SH3BP1 | 22 | 38010661 | 38082939 |
| SIPA1L1 | 14 | 71767166 | 72227946 |
| SIX3 | 2 | 45148902 | 45193216 |
| SLC10A4 | 4 | 48465360 | 48511213 |
| SLC12A5 | 20 | 44630356 | 44708784 |
| SLC17A6 | 11 | 22339643 | 22421049 |
| SLC17A8 | 12 | 100730857 | 100835837 |
| SLC18A3 | 10 | 50798347 | 50840765 |
| SLC24A3 | 20 | 19173290 | 19723581 |
| SLC32A1 | 20 | 37333105 | 37378015 |
| SLC35D3 | 6 | 137223402 | 137266777 |
| SLC4A10 | 2 | 162260843 | 162861792 |
| SLC4A3 | 2 | 220472049 | 220526702 |
| SLC4A8 | 12 | 51765101 | 51922980 |
| SLC5A7 | 2 | 108582979 | 108650450 |
| SLC6A17 | 1 | 110673108 | 110764824 |
| SLC7A14 | 3 | 170157372 | 170323863 |
| SLIT2 | 4 | 20234883 | 20642184 |
| SLITRK4 | X | 142690596 | 142743596 |
| SNAP91 | 6 | 84242599 | 84439410 |
| SNCB | 5 | 176027085 | 176077530 |
| SNPH | 20 | 1226960 | 1309972 |
| SOX11 | 2 | 5812799 | 5861516 |
| SPHKAP | 2 | 228824666 | 229066361 |
| SPTB | 14 | 65193002 | 65366601 |
| SPTBN4 | 19 | 40952148 | 41102370 |
| SRRM3 | 7 | 75811216 | 75936605 |
| SST | 3 | 187366694 | 187408187 |
| SSTR2 | 17 | 71141151 | 71187185 |
| ST8SIA3 | 18 | 54998044 | 55058962 |
| STEAP2 | 7 | 89776904 | 89887451 |
| STX1B | 16 | 30980577 | 31041949 |
| SULT4A1 | 22 | 44200389 | 44278398 |
| SUSD4 | 1 | 223374161 | 223557544 |
| SYN1 | X | 47411303 | 47499252 |
| SYN2 | 3 | 12025876 | 12252900 |
| SYNGR3 | 16 | 2019661 | 2064276 |
| SYNPR | 3 | 63193991 | 63622597 |
| SYP | X | 49024269 | 49076718 |
| SYT13 | 11 | 45241852 | 45327870 |
| SYT2 | 1 | 202539724 | 202699545 |
| SYT4 | 18 | 40827843 | 40877615 |
| SYT6 | 1 | 114611913 | 114716541 |
| TAC3 | 12 | 57383784 | 57442667 |
| TACR1 | 2 | 75253590 | 75446826 |
| TBC1D24 | 16 | 2505147 | 2575735 |
| TCEAL3 | X | 102842379 | 102904618 |
| TCEAL5 | X | 102508619 | 102551800 |
| TCEAL6 | X | 101375448 | 101417942 |
| TENM1 | X | 123489753 | 124117666 |
| TENM2 | 5 | 166691804 | 167711162 |
| TENM3 | 4 | 183045140 | 183744177 |
| TEX15 | 8 | 30669060 | 30768122 |
| THY1 | 11 | 119268090 | 119315695 |
| TMEM130 | 7 | 98455800 | 98520083 |
| TMEM132E | 17 | 32887768 | 32986337 |
| TMEM163 | 2 | 135193330 | 135496570 |
| TMEM179 | 14 | 104921015 | 105091984 |
| TMEM59L | 19 | 18698240 | 18751849 |
| TMEM91 | 19 | 41836816 | 41909988 |
| TRBC1 | 7 | 142532587 | 142574261 |
| TRBC2 | 7 | 142541934 | 142583641 |
| TRO | X | 54926895 | 54977864 |
| TSPAN18 | 11 | 44728015 | 44973972 |
| UBE2QL1 | 5 | 6428736 | 6515022 |
| UFSP1 | 7 | 100466346 | 100507339 |
| UGCG | 9 | 114639046 | 114717649 |
| UNC13A | 19 | 17692137 | 17819401 |
| UNC80 | 2 | 210616717 | 210884024 |
| UNCX | 7 | 1252543 | 1296954 |
| USP29 | 19 | 57610506 | 57663294 |
| VAMP1 | 12 | 6551403 | 6600153 |
| VAT1L | 16 | 77802427 | 78034004 |
| VAV2 | 9 | 136607016 | 136877726 |
| VGF | 7 | 100785790 | 100828874 |
| VIP | 6 | 153051933 | 153100900 |
| VSNL1 | 2 | 17700393 | 17858285 |
| VSTM2L | 20 | 36511499 | 36593752 |
| WDR6 | 3 | 49024495 | 49073386 |
| WIF1 | 12 | 65424406 | 65535346 |
| WNT5A | 3 | 55479743 | 55543973 |
| YPEL4 | 11 | 57392560 | 57437417 |
| ZCCHC12 | X | 117937753 | 117980931 |
| ZCCHC18 | X | 103336822 | 103380533 |
| ZDBF2 | 2 | 207119387 | 207199148 |
| ZFHX2 | 14 | 23970066 | 24045401 |
| ZNF23 | 16 | 71461500 | 71516998 |
| ZNF536 | 19 | 30699197 | 31224445 |

**Supplementary Table 3 |** List of Astroglial Expressed Genes, including their Chromosome and Base Pair Locations (± 20 KB)

| Gene Name | Chromosome | Start | Stop |  |
| --- | --- | --- | --- | --- |
| A2M | 12 | 9200260 | 9288825 |  |
| ABCD2 | 12 | 39923835 | 40033553 |  |
| ABHD3 | 18 | 19210858 | 19304766 |  |
| ABHD4 | 14 | 23047146 | 23101265 |  |
| ABI3BP | 3 | 100448000 | 100732359 |  |
| ACAA2 | 18 | 47289869 | 47360330 |  |
| ACAD8 | 11 | 134103389 | 134155749 |  |
| ACOT1 | 14 | 73983818 | 74030498 |  |
| ACOT11 | 1 | 54987930 | 55124865 |  |
| ACOT2 | 14 | 74014324 | 74062357 |  |
| ACSBG1 | 15 | 78439810 | 78558030 |  |
| ACSS1 | 20 | 24966868 | 25059616 |  |
| ADAMTS1 | 21 | 28188066 | 28237728 |  |
| ADHFE1 | 8 | 67322420 | 67403836 |  |
| ADRA2A | 10 | 112816790 | 112860658 |  |
| AGT | 1 | 230818269 | 230870043 |  |
| ALDH1A1 | 9 | 75495578 | 75715358 |  |
| ALDH1L1 | 3 | 125802412 | 125936837 |  |
| ALDH6A1 | 14 | 74503553 | 74571196 |  |
| APLN | X | 128759240 | 128808933 |  |
| APPL2 | 12 | 105547074 | 105650016 |  |
| AQP4 | 18 | 24412002 | 24465782 |  |
| ARHGEF26 | 3 | 153818792 | 153995616 |  |
| ATP13A4 | 3 | 193099866 | 193330900 |  |
| AXL | 19 | 41705108 | 41787671 |  |
| BMPR1B | 4 | 95659119 | 96099599 |  |
| BOK | 2 | 242478136 | 242533546 |  |
| BTBD17 | 17 | 72332555 | 72378085 |  |
| BTD | 3 | 15622848 | 15707329 |  |
| C16orf74 | 16 | 85703690 | 85804735 |  |
| C1orf198 | 1 | 230952865 | 231025335 |  |
| C4A | 6 | 31920194 | 31980852 |  |
| C4B | 6 | 32006249 | 32046275 |  |
| C4orf19 | 4 | 37435563 | 37645117 |  |
| CABLES1 | 18 | 20694528 | 20860431 |  |
| CACNG5 | 17 | 64811235 | 64901603 |  |
| CBR3 | 21 | 37487210 | 37538864 |  |
| CBS | 21 | 44453301 | 44517053 |  |
| CCDC24 | 1 | 44437031 | 44482200 |  |
| CD302 | 2 | 160605364 | 160674753 |  |
| CD38 | 4 | 15759898 | 15874853 |  |
| CD70 | 19 | 6563194 | 6624114 |  |
| CDC42EP1 | 22 | 37936454 | 37985412 |  |
| CDC42EP4 | 17 | 71259763 | 71328314 |  |
| CHRDL1 | X | 109897084 | 110059286 |  |
| CIB1 | 15 | 90753207 | 90797279 |  |
| CLDN10 | 13 | 96065858 | 96252013 |  |
| CNTFR | 9 | 34531430 | 34610121 |  |
| CPNE2 | 16 | 57106449 | 57201878 |  |
| CPQ | 8 | 97637455 | 98181882 |  |
| CSDC2 | 22 | 41936767 | 41993745 |  |
| CSGALNACT1 | 8 | 19241672 | 19635540 |  |
| CTHRC1 | 8 | 104363743 | 104415225 |  |
| CTSO | 4 | 156825270 | 156895063 |  |
| CTXN3 | 5 | 126964736 | 127014322 |  |
| CXCL14 | 5 | 134886373 | 134934969 |  |
| CYP2D6 | 22 | 42502501 | 42546908 |  |
| CYP2J2 | 1 | 60338980 | 60412462 |  |
| CYP4F2 | 19 | 15968833 | 16028930 |  |
| DAO | 12 | 109232708 | 109314819 |  |
| DBX2 | 12 | 45388455 | 45464882 |  |
| DDAH1 | 1 | 85764164 | 86063933 |  |
| DECR1 | 8 | 90993633 | 91084320 |  |
| DIO2 | 14 | 80643873 | 80874100 |  |
| DKK3 | 11 | 11964653 | 12051316 |  |
| DMRTA2 | 1 | 50863222 | 50909172 |  |
| DPY19L3 | 19 | 32876449 | 32996801 |  |
| DUSP6 | 12 | 89721009 | 89767048 |  |
| EFHD1 | 2 | 233450767 | 233567491 |  |
| EFNB2 | 13 | 107122079 | 107207462 |  |
| EGFR | 7 | 55066714 | 55344313 |  |
| ELOVL2 | 6 | 10960992 | 11064547 |  |
| EMID1 | 22 | 29581840 | 29675586 |  |
| EMP2 | 16 | 10602279 | 10694555 |  |
| EMX2 | 10 | 119281955 | 119329056 |  |
| ENHO | 9 | 34501038 | 34543039 |  |
| ENTPD2 | 9 | 139922550 | 139968497 |  |
| EPHX2 | 8 | 27328296 | 27423081 |  |
| ETNPPL | 4 | 109643196 | 109704210 |  |
| EVA1A | 2 | 75676428 | 75816848 |  |
| EZR | 6 | 159166773 | 159260444 |  |
| F3 | 1 | 94974781 | 95027356 |  |
| FABP7 | 6 | 123080620 | 123125219 |  |
| FAM20A | 17 | 66511254 | 66617530 |  |
| FGFR2 | 10 | 123217848 | 123377972 |  |
| FGFR3 | 4 | 1775034 | 1830599 |  |
| FGFRL1 | 4 | 983724 | 1040685 |  |
| FJX1 | 11 | 35619735 | 35662419 |  |
| FOXB1 | 15 | 60276421 | 60373929 |  |
| FSCN1 | 7 | 5612439 | 5666286 |  |
| FXYD7 | 19 | 35614154 | 35665204 |  |
| FZD1 | 7 | 90873783 | 90918123 |  |
| FZD10 | 12 | 130627004 | 130670285 |  |
| FZD2 | 17 | 42614925 | 42656907 |  |
| GABRA4 | 4 | 46900917 | 47016424 |  |
| GABRG1 | 4 | 46017786 | 46146098 |  |
| GAS1 | 9 | 89539279 | 89582104 |  |
| GDF10 | 10 | 48405815 | 48458976 |  |
| GFAP | 17 | 42962376 | 43014305 |  |
| GJB6 | 13 | 20776110 | 20826534 |  |
| GJC3 | 7 | 99500892 | 99547243 |  |
| GLDC | 9 | 6512464 | 6665650 |  |
| GLI3 | 7 | 41980548 | 42297469 |  |
| GNB4 | 3 | 179096990 | 179189378 |  |
| GNG12 | 1 | 68147149 | 68319150 |  |
| GNG5 | 1 | 84944008 | 84992248 |  |
| GPC5 | 13 | 92030929 | 93539490 |  |
| GPC6 | 13 | 93859095 | 95079655 |  |
| GPLD1 | 6 | 24404793 | 24515433 |  |
| GRIN2C | 17 | 72818162 | 72877627 |  |
| GSTK1 | 7 | 142921186 | 142987947 |  |
| HADH | 4 | 108890870 | 108976331 |  |
| HAPLN1 | 5 | 82913624 | 83037432 |  |
| HDAC8 | X | 71529366 | 71812953 |  |
| HES1 | 3 | 193833934 | 193876521 |  |
| HES5 | 1 | 2440184 | 2481684 |  |
| HHATL | 3 | 42714155 | 42764319 |  |
| HK2 | 2 | 75041108 | 75140486 |  |
| HOPX | 4 | 57494155 | 57568065 |  |
| HSD11B1 | 1 | 209839510 | 209928295 |  |
| HSPB8 | 12 | 119596447 | 119678936 |  |
| ID4 | 6 | 19817617 | 19860915 |  |
| IGDCC4 | 15 | 65653802 | 65735410 |  |
| IGSF1 | X | 130387480 | 130553677 |  |
| IGSF11 | 3 | 118599404 | 118884915 |  |
| IKBIP | 12 | 98987183 | 99058891 |  |
| IL18 | 11 | 111993974 | 112054840 |  |
| IRX5 | 16 | 54944774 | 54988397 |  |
| ITGB8 | 7 | 20350325 | 20475377 |  |
| ITIH3 | 3 | 52808784 | 52863025 |  |
| JAM2 | 21 | 26991584 | 27109874 |  |
| KCNJ16 | 17 | 68029570 | 68151749 |  |
| KCNN2 | 5 | 113676642 | 113852337 |  |
| KCTD5 | 16 | 2712476 | 2779031 |  |
| KLF15 | 3 | 126041478 | 126096285 |  |
| KLF3 | 4 | 38645817 | 38722663 |  |
| KLHL13 | X | 117011776 | 117271303 |  |
| LAPTM4B | 8 | 98767285 | 98885241 |  |
| LBH | 2 | 30434397 | 30566596 |  |
| LCAT | 16 | 67953653 | 67998034 |  |
| LFNG | 7 | 2532163 | 2588811 |  |
| LGI4 | 19 | 35595417 | 35653355 |  |
| LGR6 | 1 | 202143029 | 202308909 |  |
| LHX2 | 9 | 126743949 | 126815580 |  |
| LIX1 | 5 | 96407574 | 96498576 |  |
| LPAR4 | X | 77983206 | 78032591 |  |
| LRIG1 | 3 | 66409221 | 66571687 |  |
| LRRC2 | 3 | 46536913 | 46641589 |  |
| LUZP2 | 11 | 24498516 | 25124150 |  |
| LXN | 3 | 158343611 | 158410482 |  |
| MAOB | X | 43605858 | 43761693 |  |
| MAPK4 | 18 | 48066448 | 48278194 |  |
| MARCKSL1 | 1 | 32779433 | 32821980 |  |
| MBOAT2 | 2 | 8972820 | 9163942 |  |
| MDK | 11 | 46382306 | 46425375 |  |
| MERTK | 2 | 112636056 | 112807138 |  |
| METRN | 16 | 745115 | 789655 |  |
| MGST1 | 12 | 16480076 | 16782193 |  |
| MLC1 | 22 | 50477820 | 50544331 |  |
| MMP14 | 14 | 23285766 | 23338236 |  |
| MOB3B | 9 | 27305207 | 27549779 |  |
| MPV17L2 | 19 | 18283992 | 18327758 |  |
| MSI1 | 12 | 120759133 | 120826983 |  |
| MSX2 | 5 | 174131536 | 174177896 |  |
| MYBPC1 | 12 | 101942131 | 102099796 |  |
| MYOC | 1 | 171584557 | 171641823 |  |
| NAAA | 4 | 76811809 | 76882204 |  |
| NCAN | 19 | 19302782 | 19383042 |  |
| NFE2L2 | 2 | 178072323 | 178277425 |  |
| NKAIN4 | 20 | 61852136 | 61924046 |  |
| NOTCH1 | 9 | 139368896 | 139460314 |  |
| NPAS3 | 14 | 33384139 | 34293382 |  |
| NPY | 7 | 24303782 | 24351484 |  |
| NR2E1 | 6 | 108467262 | 108530013 |  |
| NRARP | 9 | 140174083 | 140216703 |  |
| NUPR1 | 16 | 28528606 | 28570495 |  |
| OAF | 11 | 120061475 | 120121041 |  |
| OMG | 17 | 29579031 | 29644557 |  |
| PACRG | 6 | 163128164 | 163756524 |  |
| PAPSS2 | 10 | 89399370 | 89527462 |  |
| PAQR6 | 1 | 156193206 | 156237881 |  |
| PAQR7 | 1 | 26167701 | 26217744 |  |
| PAX3 | 2 | 223044607 | 223183715 |  |
| PBXIP1 | 1 | 154896552 | 154948599 |  |
| PCDH10 | 4 | 134050470 | 134149356 |  |
| PCDH17 | 13 | 58185944 | 58323445 |  |
| PDLIM4 | 5 | 131573364 | 131629147 |  |
| PDPN | 1 | 13889960 | 13964452 |  |
| PHGDH | 1 | 120182421 | 120306838 |  |
| PHKG1 | 7 | 56128440 | 56180689 |  |
| PIGS | 17 | 26860401 | 26918890 |  |
| PLCD4 | 2 | 219452488 | 219521907 |  |
| PLCE1 | 10 | 95733746 | 96112580 |  |
| PLEKHO2 | 15 | 65114088 | 65180206 |  |
| PLXNB1 | 3 | 48425261 | 48491594 |  |
| PMP22 | 17 | 15113095 | 15188643 |  |
| POU3F2 | 6 | 99262580 | 99306660 |  |
| POU3F3 | 2 | 105451969 | 105496929 |  |
| POU3F4 | X | 82743269 | 82784775 |  |
| PPP1R1B | 17 | 37762993 | 37812879 |  |
| PPP1R3C | 10 | 93368199 | 93412811 |  |
| PPP1R3G | 6 | 5065720 | 5107455 |  |
| PRELP | 1 | 203424956 | 203480480 |  |
| PREX1 | 20 | 47220790 | 47464420 |  |
| PREX2 | 8 | 68844353 | 69169265 |  |
| PROCA1 | 17 | 27010215 | 27058872 |  |
| PRODH | 22 | 18880294 | 18944066 |  |
| PRRX1 | 1 | 170611869 | 170728560 |  |
| PSD2 | 5 | 139155406 | 139244051 |  |
| PSPH | 7 | 56058744 | 56139297 |  |
| PYGM | 11 | 64493861 | 64547769 |  |
| RAB31 | 18 | 9688162 | 9882548 |  |
| RAB34 | 17 | 27021299 | 27065447 |  |
| RAPGEF3 | 12 | 48108455 | 48184823 |  |
| RARRES2 | 7 | 150015408 | 150058763 |  |
| RASL11A | 13 | 27824464 | 27867827 |  |
| RBP1 | 3 | 139216276 | 139278671 |  |
| RDH5 | 12 | 56094151 | 56138489 |  |
| RFTN2 | 2 | 198412948 | 198560769 |  |
| RFX4 | 12 | 106956685 | 107176581 |  |
| RGMA | 15 | 93566636 | 93652433 |  |
| RGS20 | 8 | 54744368 | 54891863 |  |
| RHOC | 1 | 113223728 | 113270056 |  |
| RHOJ | 14 | 63650832 | 63779937 |  |
| RLBP1 | 15 | 89733100 | 89784982 |  |
| RNASET2 | 6 | 167322992 | 167390679 |  |
| RORB | 9 | 77092281 | 77328093 |  |
| S100A6 | 1 | 153487075 | 153528720 |  |
| SASH1 | 6 | 148573440 | 148893186 |  |
| SCARA3 | 8 | 27471385 | 27554293 |  |
| SCRG1 | 4 | 174285852 | 174347531 |  |
| SELENBP1 | 1 | 151316778 | 151365209 |  |
| SFRP1 | 8 | 41099481 | 41187016 |  |
| SFRP5 | 10 | 99506508 | 99551709 |  |
| SHISA9 | 16 | 12975477 | 13354272 |  |
| SLC12A4 | 16 | 67957377 | 68023504 |  |
| SLC13A3 | 20 | 45166463 | 45324714 |  |
| SLC14A1 | 18 | 43284092 | 43352485 |  |
| SLC15A2 | 3 | 121592936 | 121682949 |  |
| SLC1A4 | 2 | 65195611 | 65270999 |  |
| SLC25A18 | 22 | 18023139 | 18093760 |  |
| SLC27A1 | 19 | 17559578 | 17636977 |  |
| SLC30A10 | 1 | 219838769 | 220151989 |  |
| SLC39A12 | 10 | 17973891 | 18105286 |  |
| SLC6A11 | 3 | 10837885 | 11002419 |  |
| SLC7A10 | 19 | 33679570 | 33736756 |  |
| SLC7A11 | 4 | 139065251 | 139183503 |  |
| SLC7A2 | 8 | 17334597 | 17448082 |  |
| SLC9A3R1 | 17 | 72724791 | 72785492 |  |
| SLCO4A1 | 20 | 61253797 | 61337137 |  |
| SLITRK2 | X | 144879350 | 144927360 |  |
| SMOX | 20 | 4081627 | 4188394 |  |
| SMPD2 | 6 | 109741966 | 109785122 |  |
| SMPDL3A | 6 | 123090315 | 123150865 |  |
| SNTA1 | 20 | 31975761 | 32051698 |  |
| SOAT1 | 1 | 179242925 | 179347815 |  |
| SOD3 | 4 | 24771534 | 24822464 |  |
| SOX1 | 13 | 112701913 | 112746020 |  |
| SOX2 | 3 | 181409714 | 181452221 |  |
| SOX21 | 13 | 95341886 | 95384389 |  |
| SOX5 | 12 | 23662440 | 24123966 |  |
| SREBF1 | 17 | 17693713 | 17760325 |  |
| SRGAP1 | 12 | 64218073 | 64561613 |  |
| ST3GAL4 | 11 | 126205535 | 126330239 |  |
| ST3GAL6 | 3 | 98431080 | 98560045 |  |
| STK32A | 5 | 146594526 | 146787415 |  |
| SYT10 | 12 | 33507173 | 33612754 |  |
| TEAD1 | 11 | 12675969 | 12986298 |  |
| THRSP | 11 | 77754907 | 77799397 |  |
| TIMP4 | 3 | 12174551 | 12220851 |  |
| TLCD1 | 17 | 27031366 | 27074953 |  |
| TLR3 | 4 | 186970306 | 187029223 |  |
| TMEM176A | 7 | 150477491 | 150522208 |  |
| TMEM176B | 7 | 150468373 | 150518448 |  |
| TNC | 9 | 117762806 | 117900536 |  |
| TOM1L1 | 17 | 52956748 | 53059310 |  |
| TRIB2 | 2 | 12837015 | 12902860 |  |
| TRIL | 7 | 28972974 | 29017934 |  |
| TRPM3 | 9 | 73123979 | 74081820 |  |
| TSPAN15 | 10 | 71191229 | 71287425 |  |
| TST | 22 | 37386900 | 37435681 |  |
| TUBB2B | 6 | 3204495 | 3251964 |  |
| VCAM1 | 1 | 101165298 | 101224601 |  |
| WNT7A | 3 | 13837755 | 13941618 |  |
| WNT7B | 22 | 46296242 | 46393009 |  |
| ZFYVE21 | 14 | 104162067 | 104220005 | |

**Supplementary Table 4 | Participant Characteristics of the Irish Discovery Sample**

|  | Whole Sample | Patient Group | Control Group |
| --- | --- | --- | --- |
| *N* | 1,238 | 908 | 330 |
| Age, mean (SD) | 41.15 (12.84) | 43.02 (12.42) | 35.87 (12.64) |
| Gender (Male %) | 59.77% | 65.7% | 44.4% |
| Education, years mean (SD) | - | 12.67(2.54) | - |
| Chlorpromazine Equivalents mean (SD) | - | 461.54 (457.62) | - |
| SAPS, mean (SD) | - | 19.64 (19.19) | - |
| SANS, mean (SD) | - | 23.39 (19.86) | - |
| Full-scale IQ, mean (SD) | 100.87 (22.29) | 92.03 (19.16) | 119.91 (15.65) |
| Verbal IQ, mean (SD) | 99.14 (22.27) | 92.04 (19.99) | 118.52 (15.82) |
| Performance IQ, mean (SD) | 99.58 (18.38) | 91.11 (19.31) | 117.89 (19.17) |
| WTAR, mean (SD) | 98.53 (18.38) | 93.72 (18.75) | 109.79 (10.68) |
| LNS, mean (SD) | 8.97 (3.99) | 7.74 (3.44) | 12.45 (3.31) |
| Spatial WM, mean (SD) | -.2063 (1.500) | -.4063 (1.57) | .3197 (1.19) |
| Episodic Memory, mean (SD) | .000 (1.000) | -.3804(.8585) | 1.011 (.4454) |

Abbreviations: SAPS, scale for the assessment of positive symptoms; SANS, scale for the assessment of negative symptoms; WTAR, Wechsler test of adult reading, LNS, letter-number sequencing, WM, working memory

**Supplementary Table 5 | Participant Characteristics of the UK Biobank Sample**

| Variable | Mean (SD) | N |
| --- | --- | --- |
| Age | 56.38 (7.69) | 134,827 |
| Gender (F:M) | 71,843: 62,984 | 134,827 |
| Fluid Intelligence Score | 5.99 (2.15) | 134,827 |
| Numerical Memory Score | 6.93 (1.48) | 42,104 |
| Symbol Digit Score | 19.78 (5.04) | 44,813 |
| Microglial-SZ Polygenic Score | -7.807e-3 (5.313e-4) | 134,827 |
| Neuronal-SZ Polygenic Score | -7.206e-3 (2.654e-4) | 134,827 |
| Astroglial-SZ Polygenic Score | -5.637e-3 (3.562e-4) | 134,827 |
| Grey Matter Volume (mm^3^) | 616724.65 (55577.21) | 13,311 |

**Supplementary Table 6 |** Association between Schizophrenia Microglial Polygenic Score and Cognitive Performance in the Discovery Sample

|  | **Whole Group** | | |  | |  |  |  | | **Psychosis Patients** | | | |  | |  |  |  | **Healthy Controls** | | | | | |
| --- | --- | --- | --- | --- | --- | --- | --- | --- | --- | --- | --- | --- | --- | --- | --- | --- | --- | --- | --- | --- | --- | --- | --- | --- |
|  | *F* Change | R2 Change | β | | *p* | | | | *F* Change | | R2 Change | | β | | *p* | | *F* Change | R2 Change | | | β | | *p* |  |
| FSIQ | 6.62 | 0.008 | -0.089 | | 0.010 | | | | 1.56 | | | 0.003 | -0.052 | | 0.213 | | 0.09 | 0.00001 | | 0.022 | | 0.764 | |  |
| Perf IQ | 7.73 | 0.009 | -0.096 | | 0.006 | | | | 3.09 | | | 0.005 | -0.072 | | 0.080 | | 0.46 | 0.002 | | 0.050 | | 0.499 | |  |
| Verbal IQ | 4.78 | 0.005 | -0.070 | | 0.029 | | | | 0.82 | | | 0.001 | -0.033 | | 0.367 | | 0.50 | 0.003 | | 0.052 | | 0.481 | |  |
| WTAR | 1.84 | 0.002 | -0.047 | | 0.176 | | | | 0.04 | | | 0.0001 | -0.008 | | 0.845 | | 0.43 | 0.002 | | -0.048 | | 0.376 | |  |
| LNS | 5.47 | 0.006 | -0.075 | | 0.020 | | | | 3.20 | | | 0.004 | -0.067 | | 0.074 | | 2.80 | 0.014 | | 0.120 | | 0.096 | |  |
| Spatial WM | 2.37 | 0.003 | -0.058 | | 0.124 | | | | 0.88 | | | 0.002 | -0.040 | | 0.358 | | 1.51 | 0.010 | | 0.098 | | 0.222 | |  |
| Episodic Memory | 13.70 | 0.018 | -0.135 | | <.001 | | | | 4.84 | | | 0.009 | -0.096 | | 0.028 | | 3.68 | 0.024 | | -0.154 | | 0.057 | |  |

Abbreviations: FSIQ, full scale IQ; PERF IQ, performance IQ, WTAR, Wechsler test of adult reading, LNS, letter-number sequencing, WM, working memory

**Supplementary Figure 1 |** Genetic Analysis Pipeline


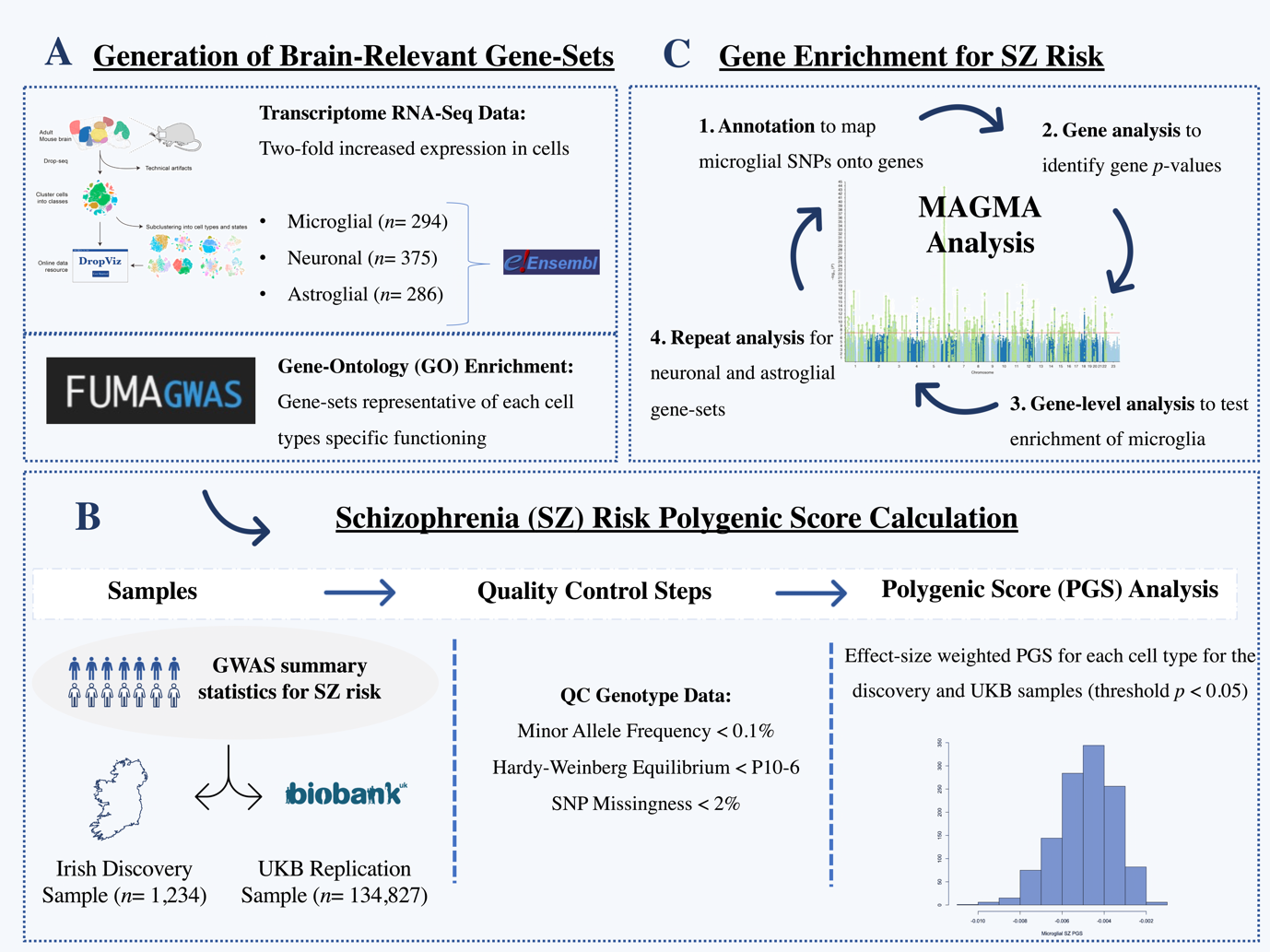


Schematic overview of the steps taken for the genetic analysis. (A) Generation of the microglial, neuronal and astroglial gene-sets with subsequent gene-ontology enrichment analysis; (B) Polygenic score calculation of the Irish Discovery sample and the UK Biobank samples. Genotype data was quality controlled and a polygenic score (based on a threshold of *p* <0.05) was calculated for each of the gene-sets in both samples; (C) Gene enrichment analysis performed for the microglial, neuronal and astroglial gene-sets to test for enrichment of these genes with risk for schizophrenia.

**References**

1. Hagenaars SP. *et al.* Shared genetic aetiology between cognitive functions and physical and mental health in UK Biobank (N= 112 151) and 24 GWAS consortia. *Mol Psychiatry*. 2016; 21(11): 1624-1632.
2. Smith A. *Symbol-Digit Modalities Test*. Los Angeles, CA: Western Psychological Services, 1991.
3. Zhang Y, Brady M, Smith S. Segmentation of brain MR images through a hidden Markov random field model and the expectation-maximization algorithm. *IEEE Trans Med Imaging*. 2001; 20(1): 45-57.
4. Alfaro-Almagro F. *et al.* Image processing and Quality Control for the first 10,000 brain imaging datasets from UK Biobank. *Neuroimage.* 2018; 166: 400-424.
5. Bycroft C. *et al.* The UK Biobank resource with deep phenotyping and genomic data. *Nature.* 2018; 562(7726): 203-209.
6. Purcell S. *et al.* PLINK: a tool set for whole-genome association and population-based linkage analyses. *Am J Hum Genet.* 2007; 81(3): 559-575.
